# Modeling policy interventions for slowing the spread of artemisinin-resistant *pfkelch* R561H mutations in Rwanda

**DOI:** 10.1038/s41591-023-02551-w

**Published:** 2023-09-21

**Authors:** Robert J. Zupko, Tran Dang Nguyen, J. Claude S. Ngabonziza, Michee Kabera, Haojun Li, Thu Nguyen-Anh Tran, Kien Trung Tran, Aline Uwimana, Maciej F. Boni

**Affiliations:** 1 Center for Infectious Disease Dynamics, Department of Biology, Pennsylvania State University, University Park, PA USA; 2 Research, Innovation and Data Science Division, Rwanda Biomedical Center (RBC), Kigali, Rwanda; 3 Department of Clinical Biology, University of Rwanda, Kigali, Rwanda; 4 Malaria and Other Parasitic Diseases Division, Rwanda Biomedical Centre (RBC), Kigali, Rwanda; 5 Department of Computer Science, Columbia University, New York City, NY USA; 6 Louvain Drug Research Institute, Université Catholique de Louvain, Ottignies-Louvain-la-Neuve, Belgium; 7 Centre for Tropical Medicine and Global Health, Nuffield Department of Medicine, University of Oxford, Oxford, UK

**Keywords:** Epidemiology, Malaria

## Abstract

Artemisinin combination therapies (ACTs) are highly effective at treating uncomplicated *Plasmodium falciparum* malaria, but the emergence of the new *pfkelch13* R561H mutation in Rwanda, associated with delayed parasite clearance, suggests that interventions are needed to slow its spread. Using a Rwanda-specific spatial calibration of an individual-based malaria model, we evaluate 26 strategies aimed at minimizing treatment failures and delaying the spread of R561H after 3, 5 and 10 years. Lengthening ACT courses and deploying multiple first-line therapies (MFTs) reduced treatment failures after 5 years when compared to the current approach of a 3-d course of artemether–lumefantrine. The best among these options (an MFT policy) resulted in median treatment failure counts that were 49% lower and a median R561H allele frequency that was 0.15 lower than under baseline. New approaches to resistance management, such as triple ACTs or sequential courses of two different ACTs, were projected to have a larger impact than longer ACT courses or MFT; these were associated with median treatment failure counts in 5 years that were 81–92% lower than the current approach. A policy response to currently circulating artemisinin-resistant genotypes in Africa is urgently needed to prevent a population-wide rise in treatment failures.

## Abstract (web summary)

A model investigating 26 possible malaria treatment policy intervention scenarios showed that introducing multiple first-line antimalarial therapies is the most effective single policy change in slowing the spread of artemisinin-resistant *pfkelch* R561H mutations in Rwanda over 5 years.

## Main

The introduction of artemisinin combination therapies (ACTs) has been instrumental in reducing the burden of *Plasmodium falciparum* malaria, but the continued evolution of drug resistance by malaria parasites has the potential to undermine these advances. Since the first appearance of artemisinin resistance in Cambodia in the 2000s^1,2^, the spread of molecular markers associated with artemisinin resistance has largely been concentrated in Southeast Asia^3,4^. However, the de novo appearance of confirmed markers for artemisinin resistance in Rwanda^5–10^ and Uganda^11,12^ signals the need for interventions to be considered in the African context^13^ to minimize the expected health, mortality and economic costs should artemisinin resistance become widespread^14^.

The ACT artemether–lumefantrine (AL) was adopted by Rwanda as the first-line therapy for uncomplicated falciparum malaria in 2006 as part of a comprehensive national strategic plan for malaria control^15^. Since adoption of AL, the *P. falciparum* kelch protein 13 (*pfkelch13*, PF3D7_1343700) R561H mutation has emerged and been validated as a marker for partial artemisinin resistance in samples collected as part of clinical drug efficacy studies between 2012 and 2015 (ref. ^3^). In contrast to the wild-type clearance rate of 2.7 h, the 561H mutant is associated with a delayed clearance half-life of 7.2 h^16^. This is similar to the clearance half-life of the 580Y mutation that emerged in Cambodia^6,8,16^. Following the original identification of 561H in the Gasabo district of Rwanda^5^, additional studies have found 561H in more districts, with recent allele frequency measurements ranging from 0.045 to 0.219 (refs. ^6,7,9,10^). These findings indicate that drug-policy interventions are now needed to delay the spread of 561H within the local *P. falciparum* population and to reduce the impact of treatment failures due to artemisinin-resistant parasites.

As of March 2023, the World Health Organization (WHO) recommends the following six ACTs for the treatment of uncomplicated *P. falciparum* malaria: AL, artesunate–amodiaquine (ASAQ), artesunate–mefloquine (ASMQ), dihydroartemisinin–piperaquine (DHA–PPQ), artesunate–sulfadoxine-pyrimethamine (AS + SP) and artesunate–pyronaridine (AS–Pyr)^17^. Within sub-Saharan Africa, the predominant therapies deployed are AL and ASAQ^18^, while the non-ACT formulation of sulfadoxine-pyrimethamine + amodiaquine (SPAQ) is commonly used for seasonal malaria chemoprophylaxis^19^. As such, sub-Saharan Africa faces a constrained drug landscape that requires national drug-policy interventions to be balanced between delaying drug-resistance evolution by the parasite—which leads to increased drug failures over the long term—and ensuring that therapies currently given are highly efficacious.

Studies detecting the mutant 561H allele indicate both increasing allele frequency and geographic spread from 2014 to 2019; however, there may still be a window of opportunity to delay or prevent the fixation of 561H in Rwanda and avert high numbers of treatment failures. Accordingly, using a mathematical model of malaria transmission, we examined 26 possible drug-policy interventions (the majority of which use existing therapies) and their ability to slow down 561H evolution and reduce long-term treatment failures. These include the replacement of the existing first-line therapy, introduction of multiple first-line therapies (MFTs) and lengthening the dosing schedule for AL from a 3-d course of treatment to up to 5 d of AL treatment in accordance with previous clinical trials^20–22^. Additionally, a more logistically complicated strategy of drug rotation is evaluated along with sequential therapy approaches (for example, 3-d AL course followed by a 3-d DHA–PPQ course) and the deployment of triple ACTs. Table 1 summarizes our findings and policy implications.

**Table 1:** Policy summary

Background	The identification of artemisinin-resistant *P. falciparum* parasites in Rwanda carrying the *pfkelch13* R561H allele requires a change to antimalarial strategy that explicitly aims to (1) slow down the spread of artemisinin-resistant genotypes and (2) minimize drug-resistance-associated treatment failures over the next decade.
Main findings and limitations	Using a stochastic individual-based *P. falciparum* transmission model calibrated to Rwanda’s malaria epidemiology and known distribution of 561H mutations between 2014 and 2019, we project that by January 2024 the 561H allele frequency in Rwanda will be between 0.29 and 0.45 (IQR). The population-level treatment failure rate is projected to surpass 10% sometime between 2024 and 2026. Lengthening the course of the currently recommended AL treatment from 3 to 5 d, or switching away from AL altogether, is projected over a 5-year span to slow down the spread of 561H and to reduce the treatment failure rate. The model projects that the most effective single policy switch is to an MFT approach with ASAQ used for 75% of treatments and DHA–PPQ used for 25% of treatments. Under this MFT strategy, the 5-year projected treatment failure rate is between 9% and 11%, whereas the status quo of using 3-d AL is projected to result in 16–20% treatment failure after 5 years.
	The major uncertainty in these evaluations is that the future evolutionary path of piperaquine resistance in Africa is unknowable. If a piperaquine-resistant phenotype emerges in Africa that has similar characteristics to the Southeast Asian phenotype, DHA–PPQ use will likely need to be restricted. If low-grade piperaquine resistance is observed in Africa, DHA–PPQ usage can be expanded if paired with real-time molecular surveillance.
	Certain next-generation approaches—such as the usage of triple artemisinin combination therapies or 6-d sequential courses of approved ACTs—have the potential to keep treatment failures low for 5 years and in some cases 10 years. Both triple therapies evaluated here and all 6-d mixed regimens of two different ACTs were projected to keep the median treatment failure percentage below 4% after 5 years.
Policy implications	These findings suggest that Rwanda’s National Malaria Control Program, and perhaps national malaria programs of neighboring countries, should consider near-term drug-policy changes specifically aimed at slowing the spread of artemisinin-resistant *P. falciparum*. MFT approaches appear to have the right balance of feasibility and efficacy in this regard. If DHA–PPQ is expected to have a prominent role in a new policy, it is imperative that DHA–PPQ deployment be paired with routine molecular surveillance for known piperaquine-resistance markers. National-level approval processes for triple artemisinin combination therapies should start early to ensure that they are available if the resistance situation worsens in the next several years. Safety and efficacy trials for sequential 6-d courses of two different ACTs should be initiated to have an additional drug-resistance-management option available if the need arises.

## Results

### Status quo

**Fig. 1:**
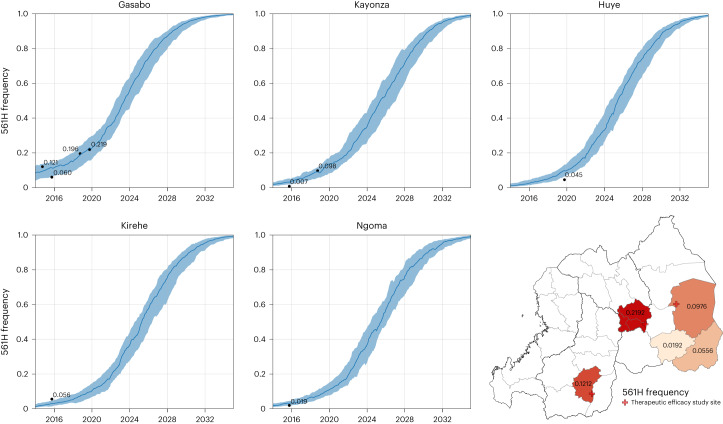
Calibration of simulated 561H allele frequency versus known frequency. The 561H mutations are artificially introduced into the simulation (10 years before detection) in the Gasabo district and allowed to evolve and increase in frequency, as shown here through model completion in 2035. During model execution, the 561H alleles spread across the simulated landscape via human migration and are selected via local drug pressure. The simulated allele frequencies in five districts (median and IQR shown with blue line and shaded area, *n* = 100) are compared to known allele-frequency data^5–7,9^ (black dots). Gasabo is a district of Kigali City; Huye district is in southern Rwanda; Kirehe, Kayonza and Ngoma districts are in eastern Rwanda. The calibrated model is largely in agreement with known 561H spatial evolutionary patterns.

**Extended Data Fig. 1:**
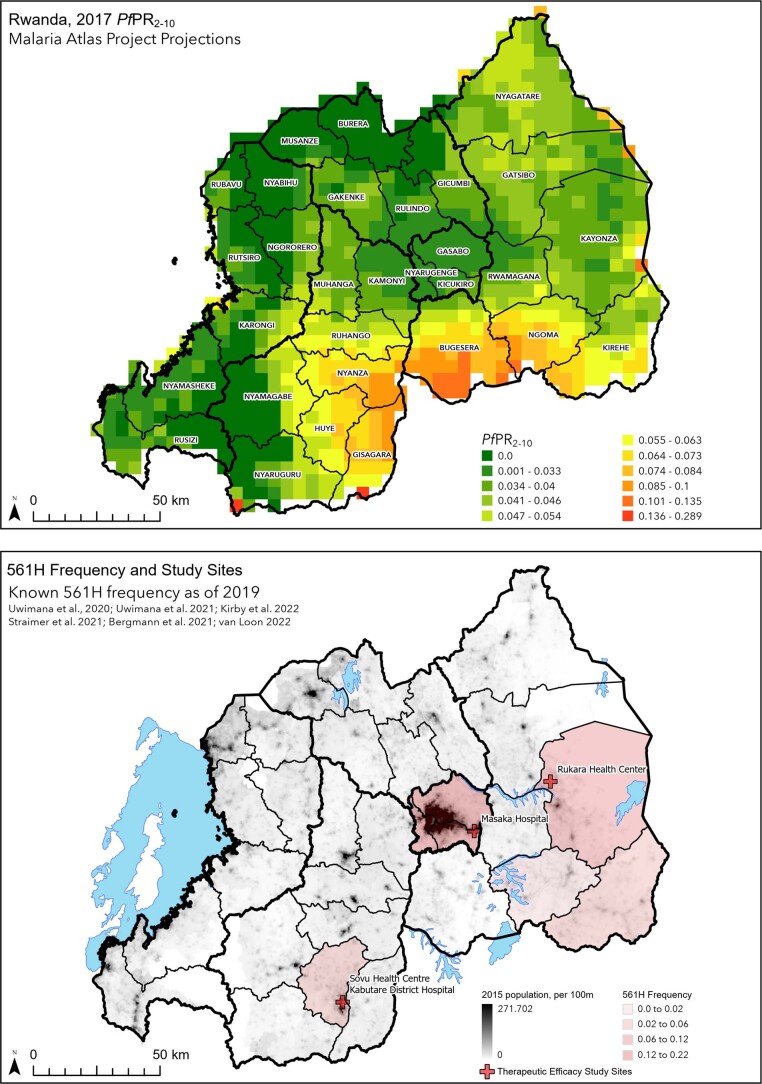
Plasmodium falciparum prevalence and allele frequency of R561H in Rwanda. **(Top)** Estimates of *P. falciparum* prevalence in 2-to-10 year-olds (*Pf*PR_2-10_) from Malaria Atlas Project^50^ where each cell is 5 km × 5 km (25 km^2^). Highest *Pf*PR_2-10_ levels above 10% are seen in the south-central part of the country. **(Bottom)** Map showing five districts in Rwanda where 561H alleles have been detected, as part of therapeutic efficacy studies (TES) or cross-sectional molecular surveys. Red crosses show sites of TESs, population is per 100 m grid cell.

To provide a common point of comparison for policy interventions, a baseline (or status quo) scenario was run (*n* = 100 replicates) in which no interventions were implemented. Our spatially calibrated model initially uses the 2017 Malaria Atlas Project (MAP) projections for Rwanda’s malaria prevalence (that is, the *P. falciparum* prevalence in ages 2–10 years (*Pf*PR_2–10_)) through the end of 2020, after which prevalence is scaled down in agreement with current incidence estimates for Rwanda^23^. Following this transmission reduction to the 2021 malaria incidence, the model presumes that incidence will remain stable over time. The allele frequency of 561H is calibrated (Fig. 1) to its measured distribution and frequency in Rwanda from 2014 to 2019 (Extended Data Fig. 1 and Supplementary Table 1). Using this calibration, the simulation forecasts that at the end of 2023, the national 561H allele frequency will be 0.36 (interquartile range (IQR): 0.29–0.45) and will reach a frequency of 0.98 (IQR: 0.97–0.99) by 2033. This projects that 561H will be effectively fixed as the dominant allele if current status quo conditions continue, a pattern typical for most but not all past patterns of antimalarial drug-resistance evolution.

The increase in 561H allele frequency is projected to be associated with an increase in treatment failures. Based on previously calibrated AL efficacy on commonly circulating genotypes in East and Central Africa^24,25^, our model estimates that in 2024, presuming no interventions, 11.15% (IQR: 9.9–12.11%) of treated *P. falciparum* malaria cases will fail treatment with the present first-line therapy of AL, or about 154,000 individuals for the calendar year (median monthly average of 12,800 (IQR: 11,200–14,000)). This monthly average is forecast to increase to 39,400 (IQR: 36,200–42,400) per month by 2033 (Supplementary Table 2). Under the current status quo conditions, the 10% treatment failure threshold recommended by the WHO for a change in first-line therapy is likely to be met or exceeded between 2024 and 2026 (12.7% median estimate for 2026, IQR: 11.2–12.9%; Supplementary Table 2).

To evaluate the impact of potential drug-policy interventions, we examined 26 national-scale drug-resistance-response strategies (Table 2), with a presumed implementation date of 1 January 2024, and calculated the relevant metrics using 3-, 5-, and 10-year endpoints, with the objectives of (1) reducing the near- and long-term numbers of treatment failures and (2) minimizing the increase in 561H allele frequency. The interventions can broadly be placed into the following five categories: (1) a change in first-line recommendation to a readily available and deployable therapy, (2) a change in strategy to deployment of MFT, (3) a change to a more intensive management approach, where multiple approaches are used sequentially with different goals at different times (for example, lowering prevalence and delaying resistance), (4) sequential dosing of two ACTs and (5) a switch to high-efficacy triple ACTs, assuming triple ACTs are approved and immediately available for emergency use.

**Table 2:** Summary of the primary drug therapy interventions examined using the simulation

Intervention	Therapy
AL extension	AL (4-d course)
	AL (5-d course)
	AL (3-d course on days 0, 1 and 2) followed up with a second course on days 7, 8 and 9; labeled ‘AL789’
AL replacement	ASAQ
	DHA–PPQ
MFTs	ASAQ (75%) + DHA–PPQ (25%)
	ASAQ (50%) + DHA–PPQ (50%)
	ASAQ (25%) + DHA–PPQ (75%)
	AL (75%) + ASAQ (25%)
	AL (50%) + ASAQ (50%)
	AL (25%) + ASAQ (75%)
	AL (75%) + DHA–PPQ (25%)
	AL (50%) + DHA–PPQ (50%)
	AL (25%) + DHA–PPQ (75%)
Sequential courses of 3-d ACT	AL on days 0, 1 and 2, followed by ASAQ on days 3, 4 and 5; labeled ‘AL, then ASAQ345’
	AL on days 0, 1 and 2, followed by DHA–PPQ on days 3, 4 and 5
	ASAQ on days 0, 1 and 2, followed by AL on days 3, 4 and 5
	DHA–PPQ on days 0, 1 and 2, followed by AL on days 3, 4 and 5
	AL on days 0, 1 and 2, followed by ASAQ on days 7, 8 and 9; labeled ‘AL, then ASAQ789’
	AL on days 0, 1 and 2, followed by DHA–PPQ on days 7, 8 and 9
	ASAQ on days 0, 1 and 2, followed by AL on days 7, 8 and 9
	DHA–PPQ on days 0, 1 and 2, followed by AL on days 7, 8 and 9
Switch to DHA–PPQ, followed by switch to MFT	DHA–PPQ (3 years), then AL (50%) + ASAQ (50%)
	DHA–PPQ (3 years), then 5-d course of AL (50%) + ASAQ (50%)
Triple ACT (TACT)	ALAQ
	ASMQ–PPQ

### Currently approved interventions

**Fig. 2:**
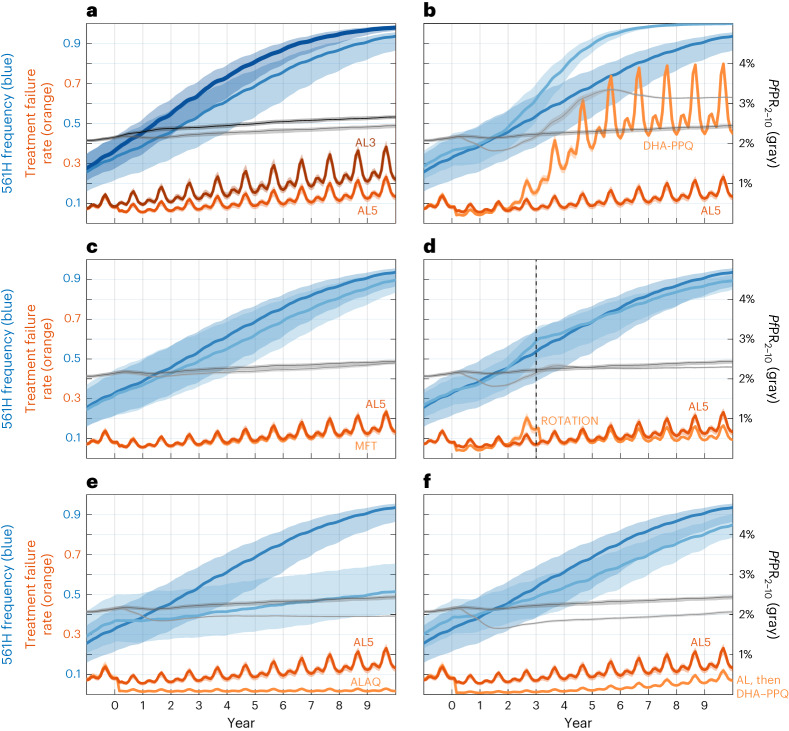
Projected 561H allele frequencies and treatment failure percentages under potential intervention scenarios. The right-hand axis shows 12-month-smoothed malaria prevalence (*Pf*PR_2–10_). All strategies are evaluated over 10 years with year 0 corresponding to the year the strategy was first implemented. Shaded bands show IQR. **a**, Comparison between the status quo treatment of 3 d of AL therapy (AL3, darker lines) and 5 d of AL therapy (AL5). **b**, Comparison between AL5 and switching to DHA–PPQ (lighter lines) as first-line therapy. Note here that under DHA–PPQ prevalence drops early (to <2%) and later rises to nearly 3.5%. This occurs because piperaquine resistance evolves quickly and reaches >0.50 genotype frequencies after 3 years, resulting in high prevalence and high levels of treatment failure. **c**, Comparison between AL5 (darker colors) and an MFT policy using ASAQ (75% of treatments) and DHA–PPQ (25%). **d**, Comparison between AL5 (darker colors) and a rotation strategy where DHA–PPQ is used for 3 years and then replaced with an MFT policy using AL5 (50%) and ASAQ (50%). **e**, Comparison between AL5 (darker colors) and the triple therapy ALAQ. **f**, Comparison between AL5 (darker colors) and sequential courses of AL on days 0, 1 and 2 and DHA–PPQ on days 7, 8 and 9.

Among alternate first-line therapies, extending the course of AL from 3 to 4 or 5 d is the most immediately available option due to stocks of AL already being present and available. Continued use of a 3-d AL course for 5 years is projected to lead to a median 561H allele frequency of 0.82 (IQR: 0.73–0.86), whereas 4-d AL results in a 561H allele frequency of 0.76 (IQR: 0.67–0.85, *P* = 0.0044; Wilcoxon rank-sum test) and 5-d AL results in a 561H allele frequency of 0.69 (IQR: 0.57–0.78, *P* < 10^−4^; Fig. 2). After 5 years, the national average monthly treatment failures are expected to be 23,500 (IQR: 20,900–26,500). These figures drop to 17,100 (IQR: 15,200–19,300, *P* < 10^−4^) under 4-d AL and 13,100 (IQR: 10,500–14,700, *P* < 10^−4^) under 5-d AL. Treatment failure numbers are improved under a longer course of AL because of the combined effect of lower 561H frequency and higher treatment efficacy of the longer course. AL efficacy generally remained high in the model’s parameterization with the majority of AL efficacies, across all genotypes in the simulation, above 85%.

Replacing AL with an alternative first-line therapy such as ASAQ or DHA–PPQ is the next most practical intervention to implement. A switch to ASAQ gives results similar to a 4-d or 5-d course of AL with a 5-year 561H allele frequency of 0.70 (IQR: 0.58–0.77, *P* < 10^−^^4^ compared to 3-d AL) and a median of 14,100 (IQR: 12,500–14,700, *P* < 10^−^^4^) treatment failures per month. However, a switch to DHA–PPQ results in an acceleration of the fixation of 561H with the allele frequency reaching 0.90 (IQR: 0.87–0.93, *P* < 10^−4^) within 5 years and fixation within 10 years (Fig. 2). The projected number of treatment failures is also high with a monthly average of 60,800 (IQR: 57,100–64,000, *P* < 10^−^^4^) after 5 years and 83,300 (IQR: 82,900–83,400, *P* < 10^−^^4^) after 10 years. When conducting sensitivity analysis for scenarios where DHA–PPQ efficacy remains relatively high, switching to DHA–PPQ is still projected to reach the 10% treatment failure threshold within 5 years of deployment, although the long-term treatment failure rates are lower (Supplementary Table 3). The rapid fixation of the 561H allele when switching to DHA–PPQ as the first-line therapy is due to the presence of artemisinin resistance coupled with the model’s projected rapid evolution of PPQ resistance leading to partner-drug failure, resulting in an environment favorable for rapid selection for artemisinin resistance.

**Fig. 3:**
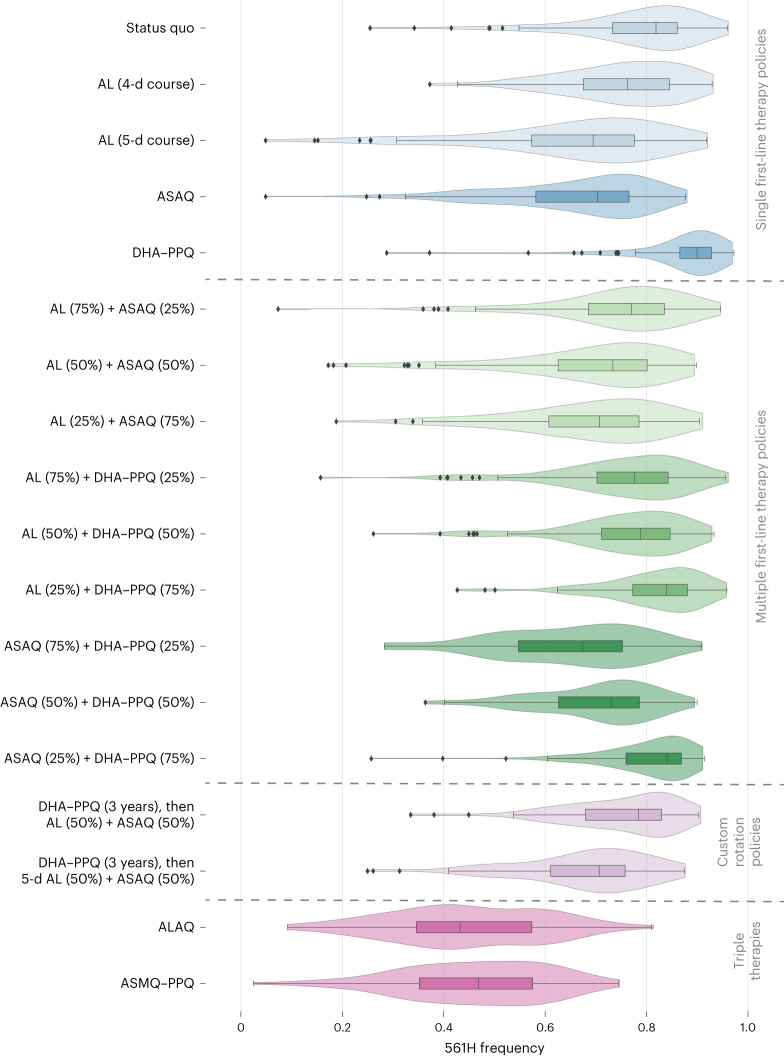
Projected 561H allele frequency after 5 years. Box plots (*n* = 100 model replicates per policy, median line with IQR) and violin plots (full data range) show the projected 561H frequency, after 5 years, under 17 policy options and the status quo of continued usage of 3-d AL. Box-plot whiskers show 1.5× IQR, and all outliers (outside 1.5× IQR) are plotted individually as diamonds. The first section includes the status quo and changes to the first-line therapies, followed by the second section containing MFT approaches, then custom drug rotation strategies and finally triple artemisinin combination therapies.

**Fig. 4:**
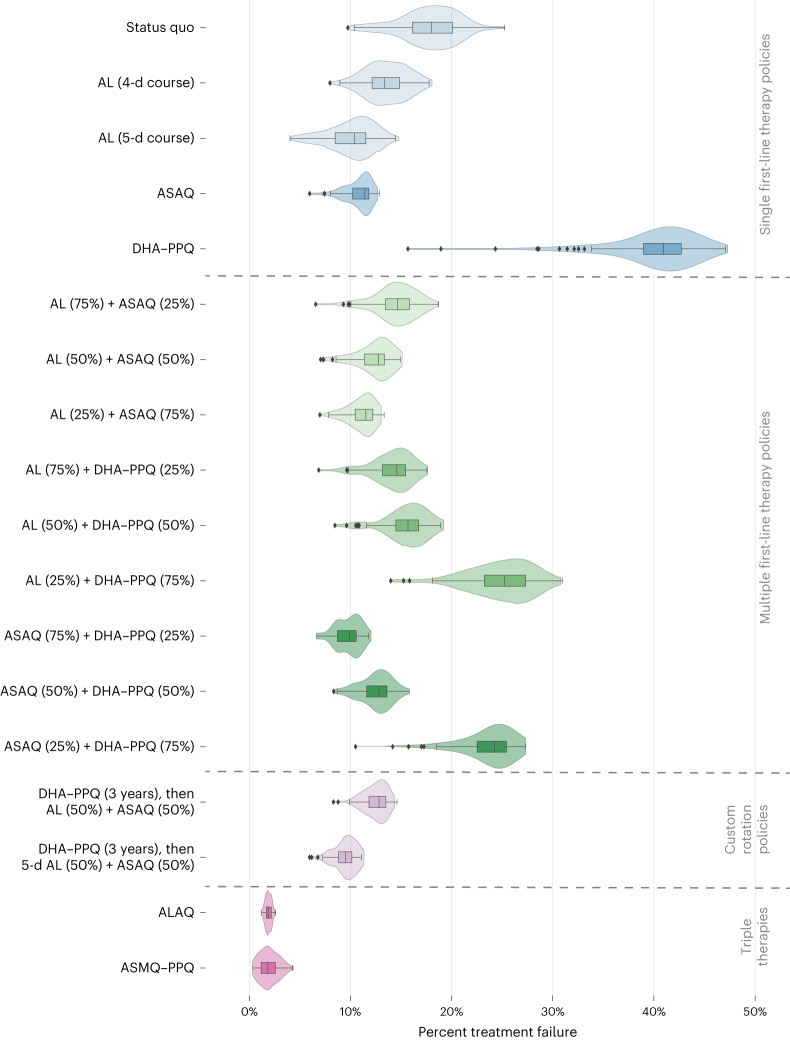
Comparison of projected treatment failures after 5 years. Box plots (*n* = 100 model replicates per policy, median line with IQR) and violin plots (full data range) show the projected population-level treatment failure rate, after 5 years, under 17 policy options and the status quo of continued usage of 3-d artemether–lumefantrine. Box-plot whiskers show 1.5× IQR, and all outliers (outside 1.5 × IQR) are plotted individually as diamonds. The first section includes the status quo and changes to the first-line therapies, followed by the second section containing MFT approaches, then custom drug rotation strategies and finally triple artemisinin combination therapies.

**Extended Data Fig. 2:**
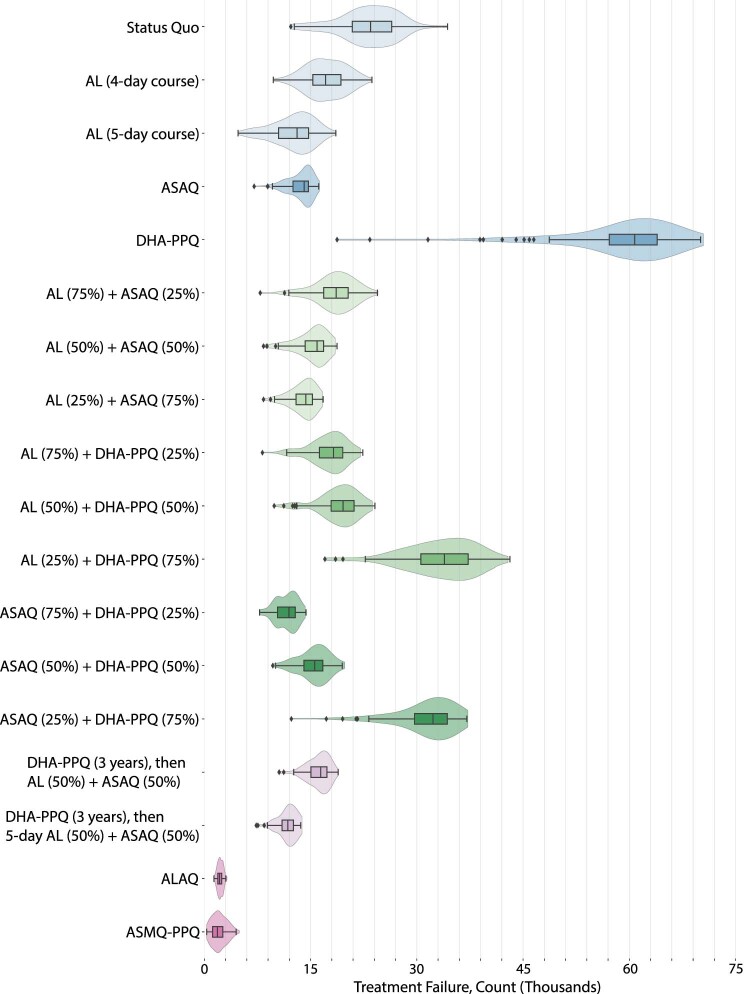
Projected monthly treatment failure counts in 2028 under various treatment strategies. Box plots (*n* = 100 model replicates per policy, median line with interquartile range (IQR)) and violin plots of treatment failure count for the same strategies as shown in Figs. 3 and 4 of main text. Whiskers show 1.5 times IQR while all outliers (outside 1.5× IQR) are plotted individually as diamonds.

In contrast to extending the duration of AL treatment or replacing the first-line therapy, the introduction of MFT will require additional logistical and operational effort, but this is likely to be offset by the effectiveness of MFT in slowing the spread of drug-resistant genotypes (due to the more complex evolutionary environment that parasites face under MFT^26–28^). Nine combinations of AL, ASAQ and DHA–PPQ with distribution ratios of 25/75, 50/50 and 75/25 were considered (Table 2) with drug choice at the time of treatment based upon a random draw in the simulation. Except for MFTs with a high (that is, 75%) proportion of DHA–PPQ treatments, MFT strategies outperformed the status quo with regard to 561H allele frequency (Fig. 3) and treatment failures (Fig. 4 and Supplementary Table 2). However, only an MFT consisting of 75% ASAQ and 25% DHA–PPQ is projected to be under the 10% treatment failure threshold after 5 years at 9.9% (IQR: 8.7–10.6%, *P* < 10^−^^4^ compared to 3-d AL). Within 10 years, all MFT approaches are projected to exceed 10% treatment failure, although combinations of 50% AL + 50% ASAQ and 25% AL + 75% ASAQ have comparably acceptable outcomes with 15.5% (IQR: 14.8–16.0%, *P* < 10^−^^4^) and 14.5% (IQR: 14.0–14.8%, *P* < 10^−^^4^) treatment failure rates, respectively. The high percentage of treatment failures in MFTs incorporating DHA–PPQ at high levels (Fig. 4) is once again due to the loss of DHA–PPQ efficacy as PPQ-resistance evolution accelerates. After 5 years, the optimal MFT policy (75% ASAQ and 25% DHA–PPQ) is projected to generate 11,900 (IQR: 10,300–12,800, *P* < 10^−^^4^) monthly treatment failures (Extended Data Fig. 2).

**Extended Data Fig. 3:**
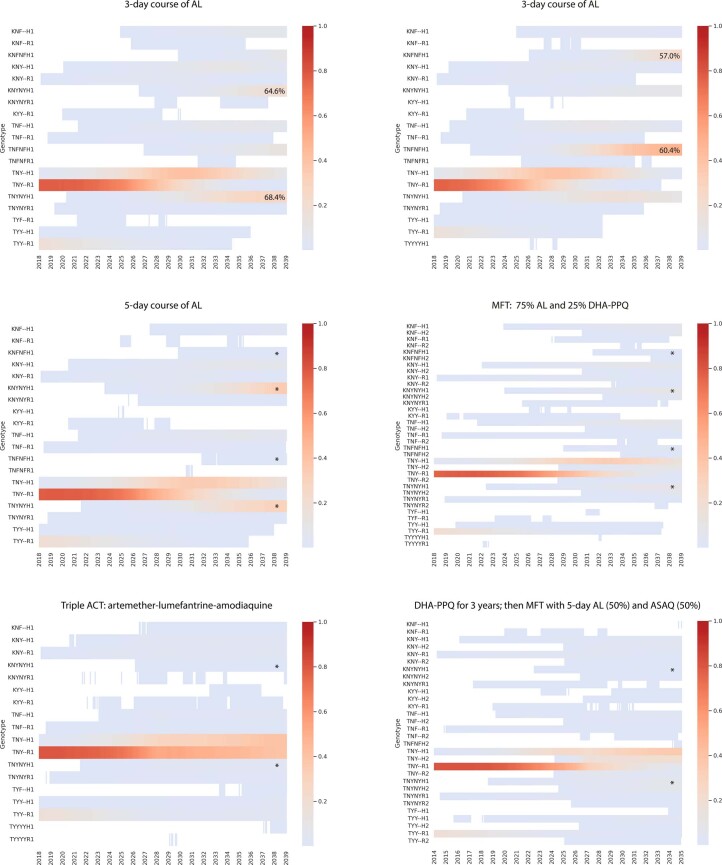
Individual genotype plots. Six heat maps showing the evolution of distinct genotypes for individual model runs. Each heat map shows genotype frequency, from 2018 to 2039, for all genotypes that rose above a 0.001 frequency at any point during this period. Frequencies below 0.001 are shown in white, whereas genotype frequencies above 0.001 are shown according to the colorbar on the right. The seven-letter genotype codes refer to, from left to right: the K76T locus, the N86Y locus, the Y184F locus, a second copy of N86Y, a second copy of Y184F, the R561H locus, and the presence (‘2’) or absence (‘1’) of a combination of piperaquine-resistant alleles. The top two heat maps show two different model runs for the status quo – differing only in that the model’s behavior is stochastic – where 3-day artemether-lumefantrine is maintained as first-line therapy. The outcomes of these runs differ, with different double-resistant (to AL) genotypes emerging and spreading. The four major AL-resistant genotypes that spread in these model runs are labeled with their efficacies in the heatmap (under a 3-day course of AL). These four double-resistant genotypes are labeled with asterisks in the four heatmaps at bottom, showing that some of the management strategies evaluated in this modeling exercise are successful at delaying the onset of AL double-resistance. For the four strategies shown at bottom, a typical model run was chosen to display. Note that under MFT, more genotypes emerge but selection pressure is weaker on each individual genotype.

While DHA–PPQ deployment, compared to all other strategies, is associated with a higher frequency of 561H over the 5- and 10-year time frame—and an associated higher level of treatment failure—over a 3-year time frame DHA–PPQ is projected to be more efficacious, suggesting that a more complex rotation strategy is worth considering. To evaluate this, we introduced the use of DHA–PPQ as the first-line therapy for 3 years, followed by a switch to an MFT using either 50% AL and 50% ASAQ or 50% 5-d AL and 50% ASAQ. These two strategies were comparable to or marginally better than pure MFT approaches. Only the strategy involving a switch to an MFT with 50% receiving a 5-d course of AL and 50% receiving ASAQ remained under the 10% threshold at the end of 5 years (9.5% (IQR: 8.8–10.1%, *P* < 10^−^^4^ compared to 3-d AL); Supplementary Table 2) with projected monthly treatment failures of 11,800 (IQR: 11,000–12,600, *P* < 10^−4^). The procurement and distribution required to deploy a drug rotation coupled with MFT suggest that compliance and operations would have a considerable influence on the success of this approach. In general, MFT strategies promote the emergence of a large number of distinct genotypes, but selection pressure is weak on each genotype, limiting their ability to reach high allele frequency (Extended Data Fig. 3).

### Next-generation interventions

**Extended Data Fig. 4:**
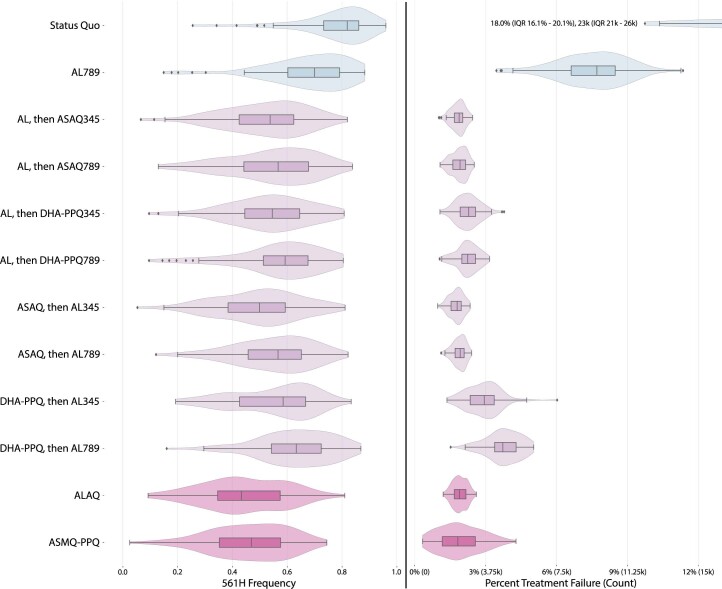
Projected 561H frequency and population-level treatment failure percentage in 2028 under sequential courses of ACT and triple ACTs. Box plots (*n* = 100 model replicates per policy, median line with interquartile range (IQR)) and violin plots of 561H frequency and percent treatment failure at a population level. Whiskers show 1.5 times IQR while all other outliers (outside 1.5 × IQR) are plotted individually as diamonds. The allele frequency is taken in December 2028 and treatment failure percentages and counts are averages over all twelve months of 2028.

We evaluated an intervention using two ACT courses sequentially for a single case of uncomplicated *P. falciparum* malaria. This approach has the advantage of an increased total dose of artemisinin (6 d), minimization of safety risks by changing partner drugs and potential exploitation of partner drugs selecting for opposite alleles. We explored this approach in the following two ways: following the protocol discussed in ref. ^29^ with six consecutive days of treatment from day 0 to day 5 inclusive, and a modified protocol in which the second course is taken on days 7, 8 and 9 (labeled as ‘789’ in Extended Data Fig. 4). This modified protocol was chosen as one that may sustain better adherence in settings where village health workers assist patients in completing their malaria treatment courses, as it allows the second course to be taken on the same days of the week as the first course. At 5 years after introduction, all sequential courses have a median 561H frequency between 0.50 and 0.63, with ASAQ followed by AL having the lowest of 0.50 (IQR: 0.38–0.59, *P* < 10^−^^4^ compared to 3-d AL). These are lower than the median 561H frequencies projected for all therapy switches, MFT approaches and rotations considered thus far. ASAQ followed by AL is also projected to have the lowest treatment failure rate 5 years after introduction at 1.8% (IQR: 1.5–2.0%, *P* < 10^−4^). All sequential courses are projected to have median treatment failure rates below 4% after 5 years and below 12% after 10 years. As in other scenarios, 10 years of DHA–PPQ use as part of a strategy of sequential ACT courses still results in strong selection pressure for PPQ resistance with long-term treatment failures increasing correspondingly (Supplementary Table 2).

While the previous 24 national-scale response strategies make use of currently available therapies, the results of past and ongoing clinical trials of the triple ACTs artemether–lumefantrine–amodiaquine (ALAQ) and artesunate–mefloquine–piperaquine (ASMQ–PPQ)^30^ suggest that they are likely to be highly efficacious and should be considered as an emergency intervention in response to rising treatment failure rates. For these two scenarios, we presume that ALAQ or ASMQ–PPQ are deployed as first-line therapy, replacing AL. As expected based upon previous modeling studies^31,32^, in our analysis, triple ACTs outperformed most other drug-policy interventions with ALAQ resulting in average monthly treatment failures of 2,100 (IQR: 1,900–2,500, *P* < 10^−^^4^ compared to 3-d AL) and a 561H allele frequency of 0.43 (IQR: 0.34–0.57, *P* < 10^−^^4^) after 5 years, while ASMQ–PPQ resulted in 1,900 (IQR: 1,200–2,600, *P* < 10^−^^4^) monthly treatment failures and a 561H allele frequency of 0.47 (IQR: 0.35–0.58, *P* < 10^−^^4^). Median treatment failure rates are projected to be 1.9% and 1.8%, respectively, after 5 years. The usage of ASMQ–PPQ comes with an increased risk of PPQ failure leading to projected treatment failures of 18.8% (IQR: 15.6–21.0%, *P* < 10^−^^4^) at 10 years after deployment. In contrast, treatment failures are likely to still be low if ALAQ is deployed reaching only 2.1% (IQR: 1.8–2.3%, *P* < 10^−^^4^) 10 years after deployment (Fig. 2).

### Patient adherence to treatment regimens

The scenarios modeled here assume that all courses of treatment will be completed in full; in practice, 100% compliance is unlikely and adherence rates may be complicated by under-dosing, over-dosing and formulation design (that is, fixed-dose combination versus copackaged blister packs), resulting in real-world compliance rates between 65% and 90% for a 3-d course of treatment^33,34^. To evaluate the possible impacts that compliance with treatment regimens would have, additional scenarios were evaluated in which ASAQ, DHA–PPQ and 3-, 4- or 5-d courses of AL were administered using low (25–70%), moderate (50–80%) and high (70–90%) compliance rates for complete courses (Supplementary Table 4). As expected, failure to comply with the prescribed course of treatment results in an increase in treatment failures; however, extended courses of AL (4 or 5 d) and ASAQ, even under low compliance, still outperform perfect compliance with a 3-d course of AL (Supplementary Table 5). No differences were seen in policy evaluation or prioritization when evaluating scenarios with imperfect compliance.

## Discussion

The projected national frequency of the 561H allele in Rwanda under a continuation of status quo treatment with a 3-d course of AL suggests that treatment failures will increase over the next 5 years and that drug-policy interventions are required to mitigate this risk as much as possible. The current spread of the 561H allele to other districts^6–10^, from its initial identification^5^, together with confirmation of 561H at frequencies similar to those projected in our simulation, suggests that the 561H allele is likely present throughout Rwanda. As recommended by the WHO, the Rwandan National Malaria Control Program (NMCP) should consider several strategies for mitigating the spread of *pfkelch13* alleles associated with artemisinin resistance^13^.

Our findings suggest that over a 5-year time frame extending the use of AL from 3 to 5 d may hold treatment failure rates at or near the 10% threshold and switching to an MFT strategy is also worth considering with the optimal MFT approach—a 75% ASAQ and 25% DHA–PPQ deployment—projected to hold treatment failure rates to 9.9% after 5 years. Extending the course of treatment with AL has minimal logistical considerations beyond ensuring sufficient quality of doses being distributed and appears to be beneficial compared to the status quo scenario. Although there are concerns regarding the cardiotoxicity of antimalarial drugs^35,36^, the incidence of adverse cardiac events recorded during clinical trials has been low^37,38^. Switching to one of several MFT options where future treatment failure rates can be kept close to 10% will ensure there are no concerns with extended artemisinin dosing, and the success of these MFT deployments will depend on operational capability around the supply and distribution of ACTs. Based on the structure of the distribution network for antimalarials in Rwanda and the current availability of different ACTs, an MFT deployment is more likely to be feasible than a custom rotation approach, although it lacks the logistical ease of a single recommended first-line therapy.

The major uncertainty in these strategy comparisons is that the future course of PPQ resistance in Africa cannot be predicted through any modeling approaches, in vitro studies or clinical trials. The DHA–PPQ-resistant lineage that emerged in Southeast Asia led to 58% treatment failure under 3 d of DHA–PPQ treatment^39^, and a number of the key mutations associated with these phenotypes are now known^40^. However, there is no guarantee that the same PPQ-resistance mutations will emerge in Africa. Any DHA–PPQ-centered strategy in Africa should be paired with routine rapid-turnaround molecular surveillance for known markers of PPQ resistance. Similarly, for lumefantrine and amodiaquine, projected scenarios of resistance evolution come with uncertainty, but the effects of currently circulating parasite mutations on the efficacies of AL and ASAQ can be estimated.

Certain new therapeutic approaches to resistance management will need specific national approval or WHO prequalification. Triple ACTs and sequential ACT courses have the advantage of higher total parasite killing than a 3-d ACT and thus an associated lower treatment failure rate. All sequential and triple ACT approaches are projected to keep treatment failure rates below 4% over a 5-year period, with no noticeable advantage seen for sequential approaches despite their higher total dose of artemisinin. A major limitation of our modeling approach is that each therapy’s pharmacodynamics and pharmacokinetics are simplified to daily killing and drug elimination rates; a more detailed analysis will be required to understand whether we should expect true differences in failure rates for these therapies. However, for both triple ACTs and sequential ACT courses, the combination of three drugs used over a short period lowers the probability of multidrug resistance emerging during a treatment course and slows down the spread of artemisinin resistance over the long term. Triple ACTs will likely enter the WHO approval process in the next year, while approval for sequential courses may have to be sought on a case-by-case basis.

To further contextualize this study within the limitations inherent in modeling studies, due to the introduction of AL in 2006, other genomic mutations, such as those associated with reduced lumefantrine susceptibility, are present in Rwanda. The introduction of mutations in the model year 2014 represents a ‘model fitting compromise’ made necessary due to the stochastic nature of the rare mutation emergence process^24,41^ from 2006 to 2014 that has not been systematically captured in any known data collections. Furthermore, while the data and model calibration indicate that 561H initially appeared in the Gasabo district, the design of the simulation renders it incapable of fitting the highly stochastic process that would have driven the initial appearance and spread of 561H. Additionally, although it is possible that the dominant strain of *P. falciparum* circulating in Rwanda may be fitness neutral^42^, the model takes a more traditional assumption and includes a fitness penalty for the 561H mutation.

Pyronaridine–artesunate deployment was not considered in our simulations as pyronaridine-resistant falciparum phenotypes have not yet been described. Assuming similar rates of emergence and similar drops in ACT efficacy due to future pyronaridine resistance, pyronaridine–artesunate would likely make a positive contribution as an addition to any of the MFT strategies shown in Table 2. The relative rankings of the different strategies are expected to stay the same; however, this would need to be confirmed with a new set of simulations. It is unknown how early we should expect to see pyronaridine-resistant parasites and whether there will be cross-resistance with other partner drugs. As with any other deployment of a new antimalarial, regular monitoring of pyronaridine–artesunate efficacy and planned in vitro resistance studies will be crucial to the early identification of resistant phenotypes.

Another major limitation for our model calibration—and any malaria modeling exercise evaluating the last 5 years of epidemiological changes—is that the effects of the COVID-19 pandemic on malaria control are not easily quantified. A mixed-methods study of three high-endemic districts in Rwanda (Gasabo, Kayonza and Rwamagana) suggests that while the distribution of malaria testing shifted, the overall decline in uncomplicated malaria cases continued through 2020 (ref. ^43^). The most recent annual case estimates for the fiscal period of July 2021–June 2022 place *P. falciparum* case numbers at around 1 million annually, with 1.16 million reported for calendar year 2021 to the WHO^44^. With these recent gains, resistance management strategies should seek to keep absolute treatment failure counts below 10,000 monthly for the medium term.

Rwanda’s NMCP’s current activities to expand indoor residual spraying activities (from 2–8 districts last decade to 12–15 districts this decade) and to introduce synergistic insecticide-impregnated nets (piloted in 2019–2020 with distribution planned for 2023 (ref. ^45^)) are likely to reduce biting rates and number of cases in the coming years. A recent pilot study in a rural part of the Gasabo district with high malaria risk showed that larviciding activity had a moderate effect on reducing incidence; the NMCP also expects to expand larviciding as part of a new set of malaria control activities. These interventions may prove beneficial in the fight against drug resistance as they may eliminate pockets of transmission, including drug-resistant alleles, and are likely to slow the overall geographic spread of all genotypes. These activities and their effects are not included in the present modeling analysis.

For the majority of national drug-policy intervention scenarios considered here that are based upon existing therapies or protocols, treatment failures are projected to exceed 10% within 5 years and may reach as high as 40% if, under a worst-case scenario, DHA–PPQ-resistant phenotypes similar to the known Southeast Asian lineages were to emerge or be imported into Africa (Supplementary Tables 6 and 7). As such, emphasis should be placed on the development of next-generation strategies along with continued evaluation of pyronaridine–artesunate, triple ACTs and sequential ACT courses. According to model projections, the most successful among these choices in slowing down the spread of 561H and reducing treatment failures is likely to be the adoption of triple ACTs following the completion of clinical trials and regulatory approval. Early introduction of triple ACTs, before crossing the WHO first-line therapy treatment failure threshold of 10%, would need to be accompanied by appropriate public health communication and an assessment of acceptability in affected communities^46^. Model projections also show that sequential ACT courses may have comparable treatment failure benefits to triple ACTs, but the prolonged course may result in lower patient adherence.

Without additional interventions targeting drug resistance or general malaria transmission, drug resistance can spread rapidly once it is established, and this could leave NMCPs with limited forewarning to respond to a rise in malaria cases and treatment failures. The identification of 561H in Uganda^12^ suggests that the spread of the 561H mutant is currently underway and that this spread is likely to co-occur with that of other markers for artemisinin resistance within sub-Saharan Africa^11,12,47^. Urgency and speed in setting a new antimalarial policy—a policy that is specifically aimed at containing the spread of artemisinin-resistant *pfkelch13* mutants—are most likely to determine the success of our response to artemisinin resistance in Africa.

## Methods

### Model description

**Extended Data Fig. 5:**
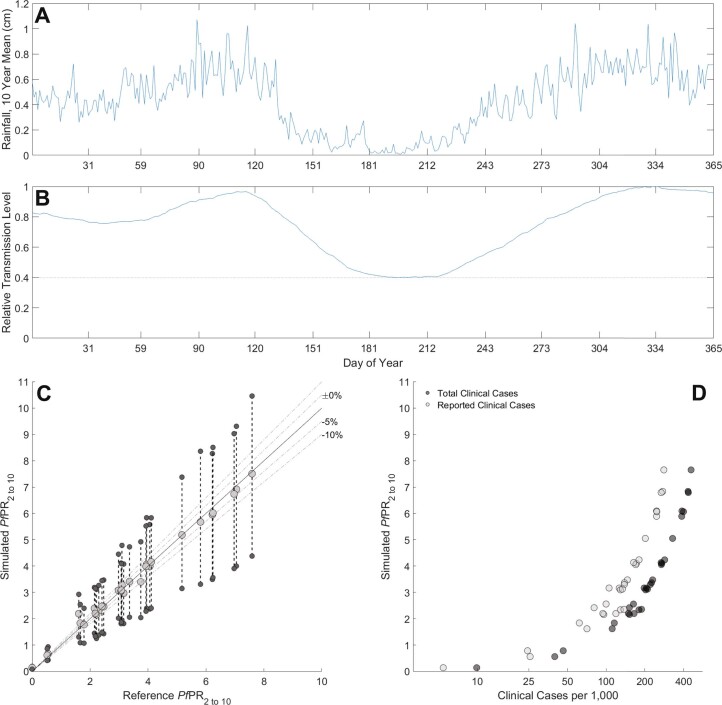
Calibration of incidence and seasonality. **(a)** Mean daily rainfall over the period 2009–2019 reported from ERA5 global climate and weather projections^64^. **(b)** Model’s relative transmission parameter (*β*) based on 54-day smoothing and normalization of rainfall data, note the lower bound of 0.4 during the low transmission season ensures some transmission is always present. **(c)** Comparison of simulated *Pf*PR_2-10_ to reference *Pf*PR_2-10_ from the Malaria Atlas Project^50^, with large gray circles showing the 12-month mean *Pf*PR_2-10_ and smaller dark gray circles showing peak and trough PfPR_2-10_ during a single year. **(d)** Relationship between model *Pf*PR_2-10_ and model’s malaria case counts (total and reported). Reported cases are total cases multiplied by the drug coverage.

A previously validated spatial, stochastic, individual-based model was used as the basis for the study^48,49^, and new model calibration and validation was performed to match the malaria prevalence of the 30 administrative districts of Rwanda. The MAP mean *Pf*PR_2–10_ projections for 2017 (ref. ^50^) were used as the basis to calibrate local transmission parameters on a 5-by-5 km (25 km^2^) scale in Rwanda (Extended Data Fig. 1), followed by a switch to the malaria incidence in 2021 using aggregate data reported by the Ministry of Health^23^. The seasonal variation in malaria transmission was coupled to seasonal rainfall (Extended Data Fig. 5a,b), consistent with the general coupling of seasonal transmission and increased rainfall. Treatment seeking and treatment coverage data were obtained from the 2019–2020 Demographic and Health Survey^51^, with treatment coverage ranging from 53.3% to 71.8% across provinces. Under the baseline model, or status quo conditions, all treated individuals in the model receive a 3-d course of AL and fully comply with the course of treatment. No private market drugs are incorporated, consistent with survey results^51^. During model execution, individuals move around the simulated landscape in a manner consistent with previous travel studies for sub-Saharan Africa^52,53^ and carry any *P. falciparum* clones to these destinations. The carried clones may then enter into circulation within a new region if the traveling individual is bitten by a mosquito, which then proceeds to infect another individual.

With these prevalence and treatment calibrations, the model produces a symptomatic malaria incidence ranging from 9.92 per 1,000 (Burera district) to 465.62 per 1,000 (Gisagara district) in 2020 under the MAP *Pf*PR_2–10_ projections, and 5.49 per 1,000 (Burera district) to 410.26 per 1,000 (Nyamasheke district) in 2022 following the switch to the incidence-based prevalence calibration. This results in an incidence of 139.13 per 1,000 for symptomatic cases and an incidence of 87.76 per 1,000 for treated cases at the national level in 2022, consistent with reporting that the incidence rate has been declining from a high of 403 per 1,000 in 2016 (ref. ^23^; Extended Data Fig. 5c,d). All policy interventions were introduced on 1 January 2024, of the simulation using version 4.1.4 of the simulation.

After calibration of prevalence, incidence and treatment coverage, the simulation’s genotype frequency and trajectory were matched to known observations of 561H allele frequencies. At the time of calibration, nine data points of the *pfkelch* 561H mutation were available at the district level—four close to the capital Kigali^5,6,9^, two from the eastern district of Kayonza^5,6^ and one each from the districts of Huye^7^, Kirehe and Ngoma^10^. To calibrate to these values, the simulation was seeded with a single mutation of 561H genotypes in Gasabo district (Kigali province) to generate a slowly growing exponential curve with district frequencies that are consistent with measured values (Fig. 1). This results in a mean national frequency ranging from 0.01 in 2014 to 0.12 in 2020. In the event that the seeded 561H genotypes went extinct before 2014, the replicate was discarded from analysis.

Because the mutation rate to 561H alleles is not known, these artificially introduced 561H genotypes are the only means by which 561H can be introduced into the simulation. The spatial spread of 561H is driven by human movement and migration within the simulation, which is based on the gravity model described in ref. ^53^ for sub-Saharan Africa and presumes that major cities (that is, Kigali) will have an oversized effect on human movement dynamics. To account for the ability of ACT partner-drug resistance to accelerate the fixation of artemisinin resistance^24^, mutations affecting other alleles (for example, *pfcrt*, *pfmdr1*, etc.) are enabled in the simulation on 1 January 2014, following the completion of model burn-in using a previously calibrated mutation rate^24^. This produces a slight model delay in *pfcrt* and *pfmdr1* mutations that are associated with the use of lumefantrine given the adoption of AL by Rwanda in 2006, likely resulting in a slight model bias toward low frequencies of alleles associated with lower lumefantrine susceptibility.

#### Model calibration and validation

The following three metrics were used for model calibration and validation: the population-weighted, district-level, annual mean *Pf*PR_2–10_ as projected by MAP^50^ versus the simulated *Pf*PR_2–10_ before 2021; the district-level clinical cases projected by the simulation and the projected 561H frequency. To calibrate the *Pf*PR_2–10_, the local transmission intensity, or *β*, was determined using a constrained parameter space search. This was performed by first binning the population in each 25 km^2^ cell using Jenks natural breaks optimization followed by scanning the domain of possible *β* values using a fixed population (that is, the bin size) along with the relevant population and climatic variables. Upon determining the possible *β* values, they were assigned to cells in the model by matching the population in each cell to the appropriate bin and assigning the calculated transmission intensity. Once this process was complete, the model was run and assessed against a target deviation of MAP *Pf*PR_2–10_ values to within ±10% (Extended Data Fig. 5c), although for low-prevalence districts, matching the MAP projections was a challenge when accompanied by a low population due to the increase in stochasticity present in the model. We found the overall *Pf*PR_2–10_ calibration to be acceptable, with low-prevalence districts having a simulated *Pf*PR_2–10_ that skewed slightly higher than the reference, whereas the higher prevalence districts skewed slightly lower.

**Extended Data Fig. 6:**
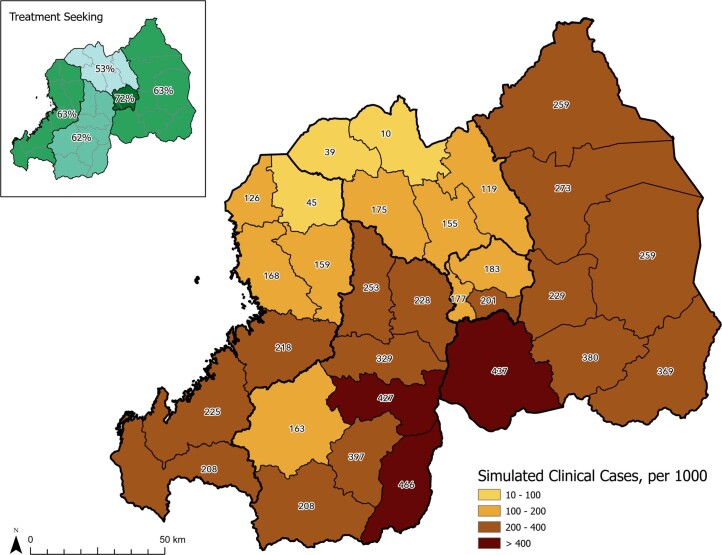
Treatment seeking by province and model incidence by district. Map shows model incidence of *P. falciparum* cases in 2020, by district. Inset shows treatment coverage, from DHS data^51^, by province.

**Extended Data Fig. 7:**
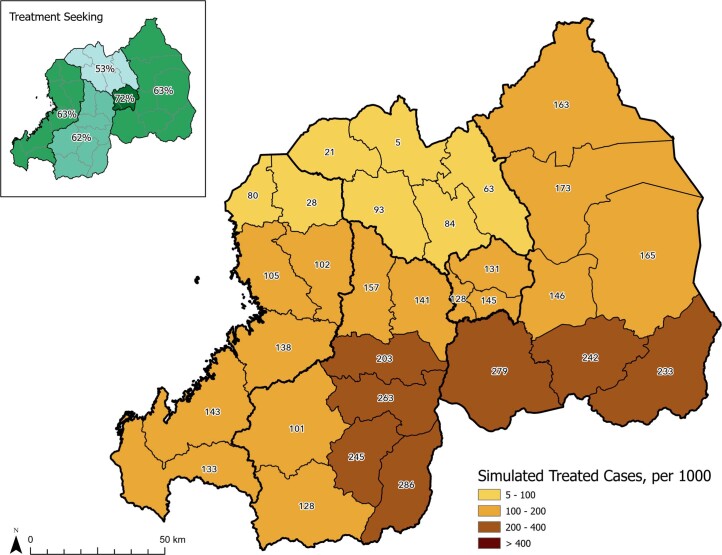
Treatment seeking by province and treated clinical cases by district. Map shows model incidence of treated *P. falciparum* cases in 2020, by district. Inset shows treatment coverage, from DHS data^51^, by province.

The next model validation metric is a comparison of the district-level projections for clinical cases versus the true incidence for 2017 (ref. ^54^). Starting with the total clinical cases per 1,000, it is clear that the spatial distribution of model-generated clinical cases is distributed in a manner that is consistent with 2017 true incidence^50^, although the counts of both all clinical cases (Extended Data Fig. 6) and treated cases (Extended Data Fig. 7) are lower in the model than in the reported figures^23^. However, these lower model projections are consistent with the overall decline in malaria cases in Rwanda^55^ and also support the simulation transition from using a calibration based on MAP projections to one based on incidence projections during model execution at the start of 2021. Overall, the good agreement between the projected *Pf*PR_2–10_ and reference values supports model calibration as being within acceptable bounds.

**Extended Data Fig. 8:**
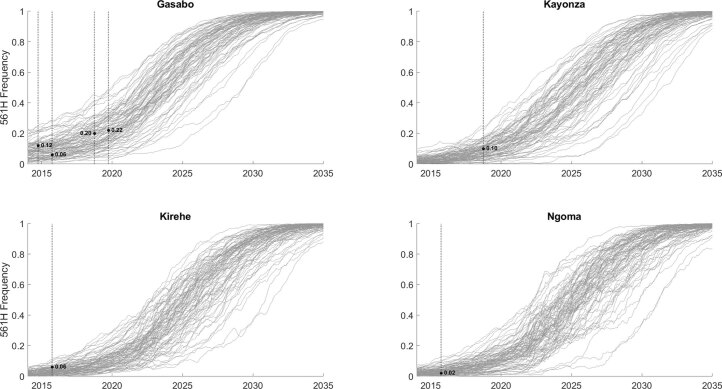
Individual trajectories of R561H evolution. Each panel shows model’s frequency of 561H allele (*n* = 100 replicates) in a particular district or province of Rwanda: Gasabo province **(Top Left)**, Kayonza district **(Top Right)**, Ngoma district **(Bottom Right)**, Kirehe district **(Bottom Left)**.

The final point of model calibration and validation is the 561H frequency. Presently, 561H frequency data are only available for Huye, Kayonza, Kirehe and Ngoma districts, along with the province of Kigali, consisting of Gasabo, Kicukiro and Nyarugenge districts (Supplementary Table 1). As a result, the model was calibrated to use a single introduction event on 30 September 2004, in which 6% of infected individuals in any of the cells of Gasabo district had their parasites switched from R561 to 561H. This date and quantity of mutations were selected on the basis of a parameter space search, which is the only introduction of 561H in the simulation. Although this introduction results in a generally consistent spread of the mutation, outliers are still possible (Extended Data Fig. 8). To ensure that replicates used for analysis are in good agreement with observed data, only replicates in which the 561H frequency in Gasabo is greater than 0.01 in September 2014 of the simulation are retained. The model’s spread of mutations is consistent with measured allele frequencies between 2014 and 2019 (refs. ^5–7,9^), supporting the model as having a 561H introduction that is properly calibrated. As a note of caution, although this calibration suggests a mechanism through which 561H may have spread in Rwanda, the model was not designed, configured or calibrated to explore the nature of the introduction event or original mutation event.

#### Model scenarios

As a common point of comparison, a baseline scenario was run in which the calibrated model was simulated for a 10-year window past the proposed point of intervention (that is, 1 January 2024 to 1 January 2034). This baseline scenario also controls for any deviations in the model versus real-world data for Rwanda by allowing all policy interventions to be compared to the same projected outcome and presumes that the calibrated malaria incidence remains stable over time. A total of 26 drug-policy intervention scenarios were evaluated within the simulation (Table 2), and all interventions were introduced at the same time with no delay between the introduction and the change in therapies received by individuals. The 5-year endpoints are presented in the main text, with 3- and 10-year endpoints referred to as needed; comprehensive results for all endpoints can be found in Supplementary Tables 2 and 5.

The policy scenarios run may be summarized as follows. Five evaluated interventions involved a change in first-line therapy to a 4-d course of AL, 5-d course of AL, ASAQ and DHA–PPQ. Nine of the interventions involved MFT approaches with drug distribution proportions ranging from 25/75 to 50/50 to 75/25 (Table 2), with selection of the drug given to the individual based upon a random draw upon first treatment. Functionally this can be imagined working the same as a provider randomly assigning a therapy to a patient via coin flip, randomization schedule or other means. Other MFT implementations with different approaches to drug distributions are possible^28^ but were not evaluated here. Two interventions considered drug rotation with short-term DHA–PPQ use first (for 3 years) followed by replacement of DHA–PPQ with one of two MFT strategies using AL and ASAQ. Four interventions replicated the therapeutic arms of the sequential ACT therapy regimen proposed in ref. ^29^ with AL followed by either ASAQ or DHA–PPQ, or AL preceded by ASAQ or DHA–PPQ. However, the model scenario deviates from the protocol proposed in ref. ^29^ by applying sequential therapy to all treatment-seeking individuals as opposed to just children aged 6–120 months. The second of the sequential therapies was given either on treatment days 3, 4 and 5 or on treatment days 7, 8 and 9; results for both timings of treatment courses are presented. Finally, two interventions considered the replacement of AL with one of two triple therapies (ALAQ and ASMQ–PPQ), which are yet to be approved^30,46^.

#### Sensitivity analysis

Five forms of sensitivity analysis were performed using the same base calibration previously described, with the relevant parameters adjusted as needed. These studies included assessing the sensitivity to individual movement, the fitness cost associated with drug-resistance mutations and the impact of individual compliance with drug treatments. All studies used at least 100 replicates per permutation in assessing the results.

First, the model sensitivity to individual movement was assessed by adjusting the movement to be 0.3, 0.5, 2 or 3 times more than the calibrated movement. Under these conditions, it was found that a movement rate less than the calibrated value resulted in projected 561H frequencies that were higher than observed in Gasabo while lower in other districts (Supplementary Figs. 1 and 2). However, higher movement rates (that is, 2× and 3×) have a lower variance and plausibly reproduced the observed 561H frequencies for Gasabo, Kayzona, Kicukiro and Nyarugenge districts, although the projected frequencies were substantially higher for Huye (Supplementary Figs. 3 and 4). These results show that the calibrated individual movement rate is consistent with the observed geographical frequency of 561H, although it is possible that the true individual movement rate may be slightly faster.

**Extended Data Fig. 9:**
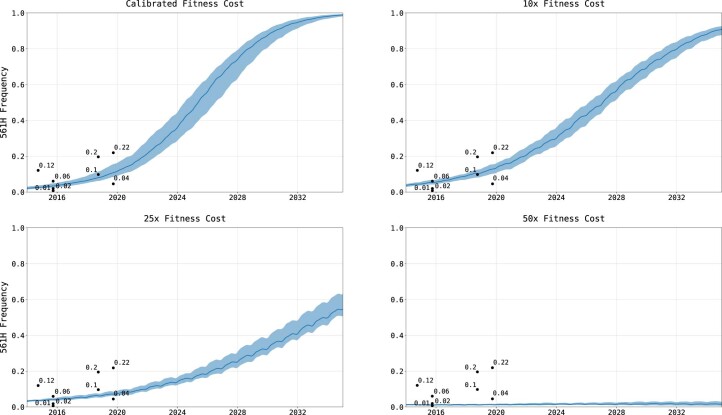
National 561H allele frequency under different assumptions on fitness cost of resistance. Model trajectories show median, national 561H allele frequency (shaded area is 95% range) under previously accepted fitness cost assumptions^24,32,48,49^ which result in a 17% within-host fitness cost per year. Sensitivity analysis increasing the daily fitness cost 10-fold **(Top Right)**, 25-fold **(Bottom Left)**, and 50-fold **(Bottom Right)** show that the fitness cost used in our simulations is consistent with allele frequency measurements (black dots) taken between 2014 and 2019. A 10-fold larger fitness cost is also compatible with the data post-2014, but it is unknown which of these values is compatible with the unseen emergence pattern from prior to 2014.

Next, the model sensitivity to fitness cost was assessed by adjusting all fitness costs to be 10, 25 or 50 times greater than previously calibrated values. Given that there is evidence that there is a 561H mutant strain of *P. falciparum* circulating in Rwanda that does not incur a fitness penalty^42^, lower values for the fitness cost were not assessed. Under the 10× scenario, it was found that the initial mutation event needed to be increased to 20% of the infected individuals in the Gasabo district, but the 561H frequency was similar to the observed data and the projection was similar to the model calibration (Supplementary Fig. 5). This suggested that the calibrated fitness penalty used in the model, based upon previous modeling exercises^24^, is reasonable and the model projections would still remain valid when the actual fitness cost incurred by the 561H mutant is up to 10× the calibrated value. However, when increasing the fitness penalty to 25×, it is necessary to increase the initial mutation event to be 50% of the infected individuals in the Gasabo district (this is unrealistic) and the allele frequency trajectories only weakly tracked the observed data points (Extended Data Fig. 9 and Supplementary Fig. 6). Under the 50× scenario, the initial mutation event was increased to 75% of the infected individuals in the Gasabo district and the projected frequencies suggest that 561H would only exist in Rwanda at a low frequency, or would soon face extinction (Supplementary Fig. 7). It is highly unlikely that higher fitness costs of 25× or 50× are plausible.

The model sensitivity to individual drug treatment compliance was assessed by administering ASAQ, DHA–PPQ and 3-, 4- or 5-d courses of AL to individuals using low (25–70%), moderate (50–80%) and high (70–90%) compliance rates for complete courses (Supplementary Table 4). Each scenario takes effect on 1 January 2024, and presumes that the individual will always complete the first day of treatment, and compliance rates then proceed to drop after that point. As expected, across all treatment options, failure to comply with the prescribed course of treatment results in higher treatment failures when compared to the full compliance outcomes. Under the 3-, 4- and 5-d AL treatment options, along with ASAQ, the number of treatment failures increases with the rate of noncompliance.

Because PPQ resistance may evolve differently in Rwanda than what was observed in Southeast Asia, the model’s sensitivity to PPQ resistance was evaluated by first evaluating the sensitivity to the gene duplication rate for *plasmepsin 2/3* genes. Using the calibrated value based upon the emergence of PPQ resistance in Southeast Asia as the baseline^25^, the probability of gene duplication was adjusted to be 0.25, 0.5, 1.25 and 1.5 times the calibrated value. These represent optimistic scenarios in which African strains of *P. falciparum* are less likely to develop PPQ resistance (0.25× and 0.5× scenarios) and pessimistic scenarios in which African strains are more likely to develop PPQ resistance (1.25× and 1.5× scenarios). For all four scenarios, the annual percentage of treatment failures exceeds the 10% treatment failure threshold within 5 years after switching from AL to DHA–PPQ (Supplementary Table 3). Ten years after the introduction of DHA–PPQ, the annual percentage of treatment failures was approximately 55% with 561H fixed for all scenarios, indicating that PPQ efficacy is a critical value to monitor.

Finally, the sensitivity of the model to changes in PPQ efficacy on PPQ-resistant parasites was evaluated by altering the configured EC_50_ value (concentration at which killing is 50% of maximum). The details of the pharmacokinetic/pharmacodynamic model are described in ref. ^48^, and the model uses calibrated values of 0.58 for PPQ-sensitive and 1.4 for PPQ-resistant parasites, where 1.0 is set as the standard therapeutic dose given in the simulation. The simulation was then run with values ranging from 1.0 to 1.6 for the EC_50_ of PPQ. As expected, an EC_50_ value less than the configured value of 1.4 had fewer treatment failures at 5 years while 1.5 and 1.6 produced more treatment failures (Supplementary Table 3). This pattern was similar for the 561H frequency, although the difference was only statistically significant for EC_50_ ≤ 1.2 with *P* < 10^−4^. For all scenarios, the model projects that the 10% treatment failure threshold would still be reached within 5 years of DHA–PPQ introduction and 561H would be fixed within 10 years.

#### Statistical analysis

Differences between groups of simulation results (that is, comparing 100 simulations each from two scenarios) are tested with nonparametric Wilcoxon rank-sum tests, and all *P* values lower than 10^−4^ are shown as 10^−4^.

### Model calibration data

#### Spatial data

A 5-by-5 km (25 km^2^) cell is the primary spatial unit in the simulation, the same size as the resolution of the MAP *Pf*PR_2–10_ projections used as part of the model calibration^50^. This cellular resolution results in a modeled space of 979 cells covering 24,475 km^2^ or about 93% of the total area of Rwanda (Extended Data Fig. 1a). Cells that are predominately water are not simulated, and some discrepancy in area is due to clipping along national borders. During model initialization, spatial data in the form of national districts, population, access to treatment and the beta (that is, transmission parameter) for each cell are loaded. Administrative and geographic boundaries from the World Bank^56,57^, the Rwanda National Institute of Statistics^58^ and the World Database on Protected Areas^59^ were used in the preparation of model inputs (Fig. 1 and Extended Data Figs. 1, 6 and 7).

#### Demographics, mortality and treatment seeking

The estimated population of Rwanda was 12,663,116 in 2020 with a crude birth rate of 28.8 per 1,000 (ref. ^60^), and the population skews younger with about 54.3% being under the age of 20 years^61^ (Supplementary Table 1). Deaths due to malaria continue to have a substantial impact on the mortality rates within Rwanda^62^, and the mortality rate applied to the population was adjusted to remove the deaths that were attributed to malaria using the UN population projections^63^ (Supplementary Tables 2 and 3). In the event an individual reaches the age of 100 years, they are removed from the simulation. When individuals are infected with the parasite, upon exhibiting clinical symptoms, they seek treatment based upon the surveyed treatment-seeking behavior for the province that contains the cell they are currently in with no distinction between under-5 and over-5 treatment-seeking rates^51^ (Supplementary Table 4).

#### Seasonal transmission

To account for the seasonal variation in transmission intensity, a seasonality calibration was conducted, and the results were applied to all cells during model execution. This seasonality calibration is based upon the correlation between rainfall and the *Anopheles* mosquito prevalence and presumes that transmission will begin to increase as favorable conditions increase. Thus, the transmission intensity is adjusted up, or down, by applying the seasonal adjustment value (Extended Data Fig. 5b). This adjustment was calculated by first calculating the 10-year daily average rainfall for Rwanda, using data from ERA5 global climate and weather projections^64^. The rainfall data were then clipped to the borders of Rwanda using Google Earth Engine for January 2009 to December 2019, inclusive, and then smoothed and shifted by 10 d using a MATLAB script. The 10-d offset was selected on the basis of the *Anopheles gambiae* lifecycle. Finally, a lower bound of 0.4 was used to ensure that some malaria transmission is always present at a rate consistent with seasonal patterns. The calculated adjustment is then imported during model initialization and applied during execution.

#### Drug efficacy

As part of the model calibration and validation, the drug efficacies were used as previously calculated^25^ following verification given the Rwandan population distribution. Within the current simulation, a population of 50,000 individuals, with a population distribution consistent with that of Rwanda, was infected with *P. falciparum* infections. Following transition of the infection from the liver stage to the blood stage (that is, clinical symptoms), the relevant therapy was given to the individual and the efficacy was assessed using the parasite density at 28 d after the first treatment. Individuals with a parasite density less than 10 ml^−1^ of blood were counted as cleared (failed otherwise). Drug efficacies of longer courses—for example, 5-d AL or two sequential consecutive courses of ACT—are calculated via a one-compartment pharmacokinetic model and traditional Hill-function pharmacodynamic model, using a 1-d time step^48^.

The lowest efficacy in the simulation is that of DHA–PPQ on the 561H genotype, which also carries PPQ resistance (43.3% efficacy); this is also the reason that PPQ resistance spreads so quickly in the model simulations. The efficacy of 3-d AL on wild-type *P. falciparum* is 95.5%, while the efficacy of 5-d AL is 97.5%. The complete drug efficacies are included in Supplementary Tables 5 and 6 and were within ±2.5% of the original efficacies calibrated in ref. ^25^.

#### Ethics and inclusion in global research

This study included researchers from the Rwanda Biomedical Research Center (who were colocated and in close collaboration with Rwanda’s NMCP). Under the leadership of A. Uwimana and C. Ngabonziza, they planned the major study-design aspects for this paper and assisted the mathematical modeling team in identifying drug-resistance-management strategies that could be feasibly implemented in Rwanda. Research roles and responsibilities were discussed throughout 2022, including during in-person meetings in August and October 2022. Capacity co-enhancement collaborations have already begun by planning (1) a local modeling instance to be run in RBC Kigali with M. Kabera as the primary analyst and (2) a series of tutorials for Penn State scientists to understand the operational aspects of drug delivery and distribution in Rwanda.

### Reporting summary

Further information on research design is available in the Nature Portfolio Reporting Summary linked to this article.

## Online content

Any methods, additional references, Nature Portfolio reporting summaries, source data, extended data, supplementary information, acknowledgements, peer review information; details of author contributions and competing interests; and statements of data and code availability are available at 10.1038/s41591-023-02551-w.

### Supplementary information

Supplementary Information Supplementary Tables 1–4 (concerning calibration parameters) and Figs. 1–7 (concerning model sensitivity analysis).

Reporting Summary

Supplementary Tables Supplementary Table 1: 561H frequency, published studies; Supplementary Table 2: primary study results; Supplementary Table 3: piperaquine sensitivity studies; Supplementary Table 4: compliance rates for sensitivity analysis; Supplementary Table 5: results for sensitivity to compliance rate; Supplementary Table 6: drug efficacy primary studies; Supplementary Table 7: drug efficacy compliance studies.

## Figures and Tables

**Extended Data Table 1:**
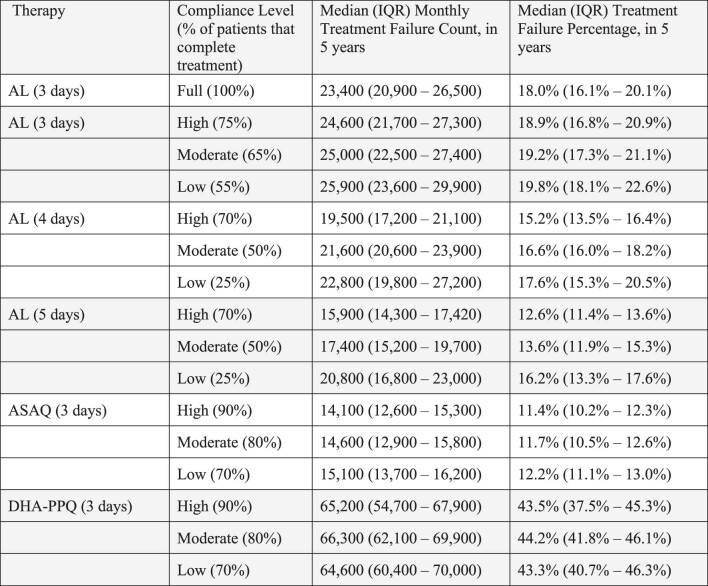
Effects of treatment compliance on treatment failure counts and percentages in 5 years

## Data Availability

Intermediate data files produced by the simulation can be found on GitHub at https://github.com/bonilab/malariaibm-spatial-Rwanda-561H/tree/main/Data. The configuration files used for the study described in this paper can be found at https://github.com/bonilab/malariaibm-spatial-Rwanda-561H/tree/main/Studies. The prevalence data for 2017, released by the MAP (version 2019), has been archived under https://github.com/bonilab/malariaibm-spatial-Rwanda-561H/tree/main/Data/GIS/MAP. The spatial distribution of population is derived from the WorldPop 2015 spatial distribution of population in Rwanda and can be found at https://hub.worldpop.org/doi/10.5258/SOTON/WP00674.

## Code availability

The archived version of the simulation code base, complied binaries for Linux and scripts used for analysis and production of figures presented in this paper can be found on GitHub at https://github.com/bonilab/malariaibm-spatial-Rwanda-561H.
