# Supplementary material for: Modeling policy interventions for slowing the spread of artemisinin-resistant pfkelch R561H mutations in Rwanda
Source: Nat Med. 2023 Sep 21;29(11):2775–84. doi: 10.1038/s41591-023-02551-w (PMC10667088; doi:10.1038/s41591-023-02551-w)

nature medicine

Article

https://doi.org/10.1038/s41591-023-02551-w

Modeling policy interventions for slowing the spread of artemisinin-resistant *pfkelch* R561H mutations in Rwanda

In the format provided by the authors and unedited

Supplemental Figure sand Tables for "Optimal drug policy interventions for slowing the spread of the *pfkelch* R561H artemisinin-resistance mutation in Rwanda", Zupko et al. (2023)

Table S1. Population distribution of Rwanda (Malaria and Other Parasitic Diseases Division of the Rwanda Biomedical Center Ministry of Health [Rwanda] & ICF, 2018)

Age Band	Population	Age Band	Population
< 5	15.1	45 – 49	3.6
5 – 9	14.1	50 – 54	3
10 – 14	14.5	55 – 59	3.1
15 – 19	9.6	60 – 64	2.2
20 – 24	7.3	65 – 69	1.2
25 – 29	6.9	70 – 74	0.9
30 – 34	6.5	75 – 79	0.6
35 – 39	6.1	80+	0.8
40 – 44	4.5		

Table S2. Proportion of deaths that are attributable to malaria for 2012 to 2018 (Republic of Rwanda, Ministry of Health, n.d., p. 82)

	2012	2013	2014	2015	2016	2017	2018
< 5 due to Malaria	48	64	173	130	158	139	84
< 5 all deaths	1647	1854	5080	4385	4162	3842	3802
> 5 due to Malaria	314	401	416	401	685	244	241
> 5 deaths	7379	7275	4356	7175	7846	5464	6249
< 5 malaria proportional	0.029	0.035	0.034	0.03	0.038	0.036	0.022
> 5 malaria proportional	0.043	0.055	0.096	0.056	0.087	0.045	0.039

Table S3. Malaria adjusted mortality rate used in the simulation, derived from UN population projections (UN, 2019).

Age Band	Mortality Rate
0 – 1	0.02641
2	0.00202
3	0.00202
4	0.00202
5	0.00198
6	0.00247
7	0.00247
8	0.00247
9	0.00247
10	0.00247

Age Band	Mortality Rate
11	0.00247
12 – 15	0.00247
16 – 20	0.00455
21 – 60	0.00455
61 – 100	0.05348

Table S4. Treatment seeking behavior in Rwanda (National Institute of Statistics of Rwanda et al., 2020).

Province	Treatment Seeking
Northern Province	53.3%
Eastern Province	63.4%
Kigali City	71.8%
Southern Province	61.6%
Western Province	63.3%

Figure S1. Projected 561H frequency when human movement is 0.3x the calibrated rate.

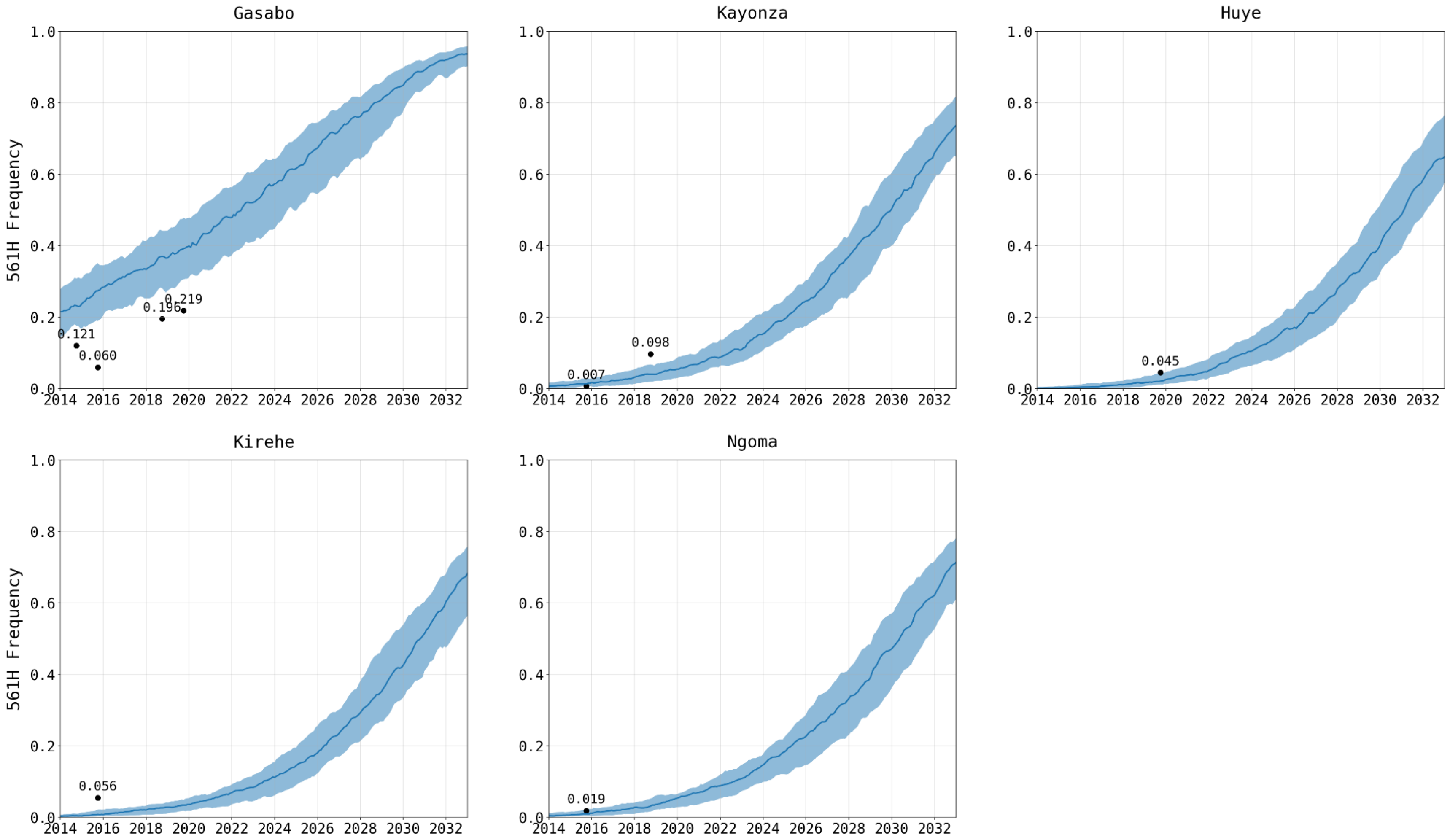

Figure S2. Projected 561H frequency when human movement is 0.5x the calibrated rate.

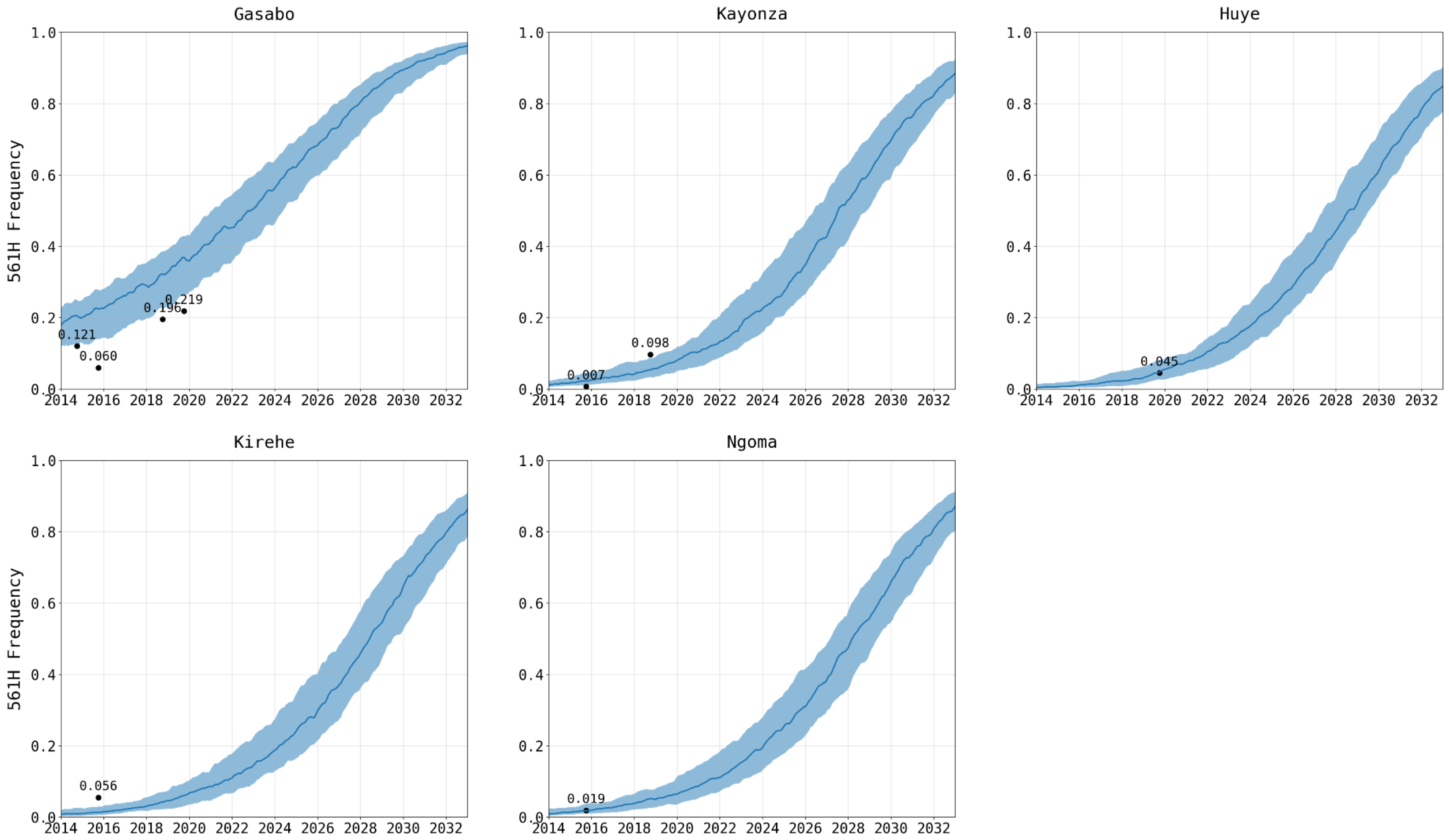

Figure S3. Projected 561H frequency when human movement is 2x the calibrated rate.

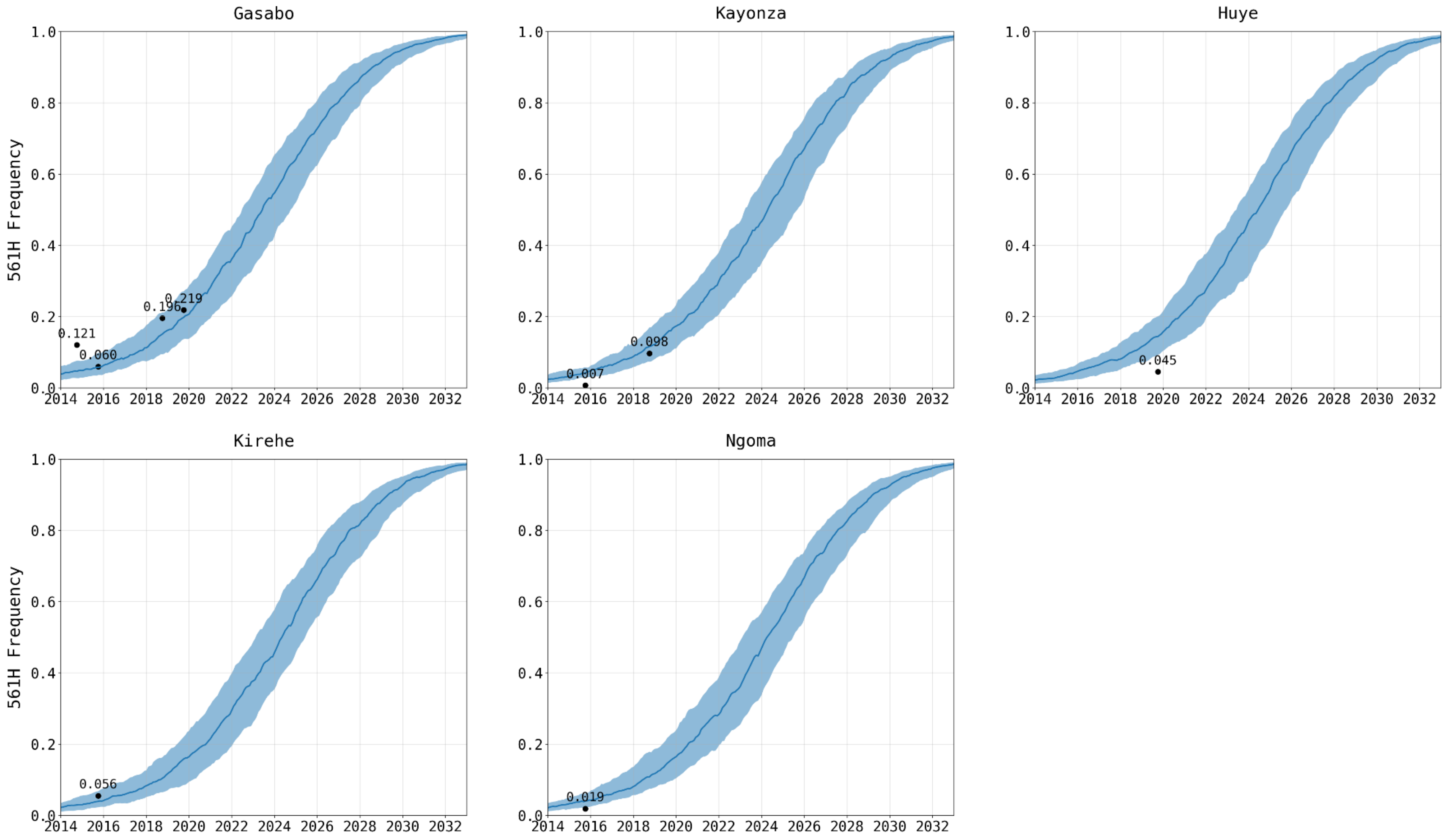

Figure S4. Projected 561H frequency when human movement is 3x the calibrated rate.

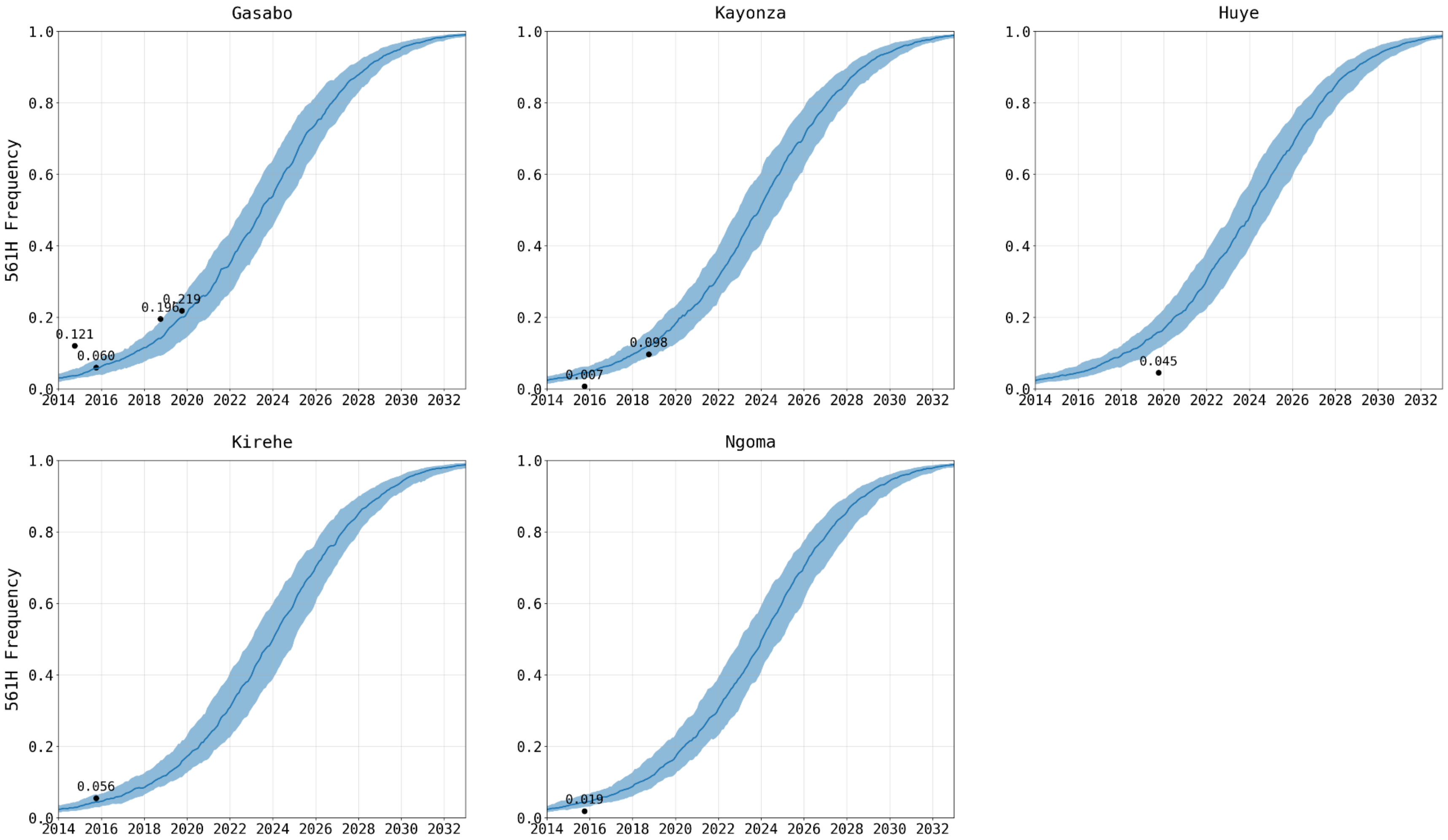

Figure S5. District level 561H frequency when the fitness penalty is increased to 10x the calibrated value and the introduction of 561H was through the one-time mutation of 20% of the infected individuals in Gasabo.

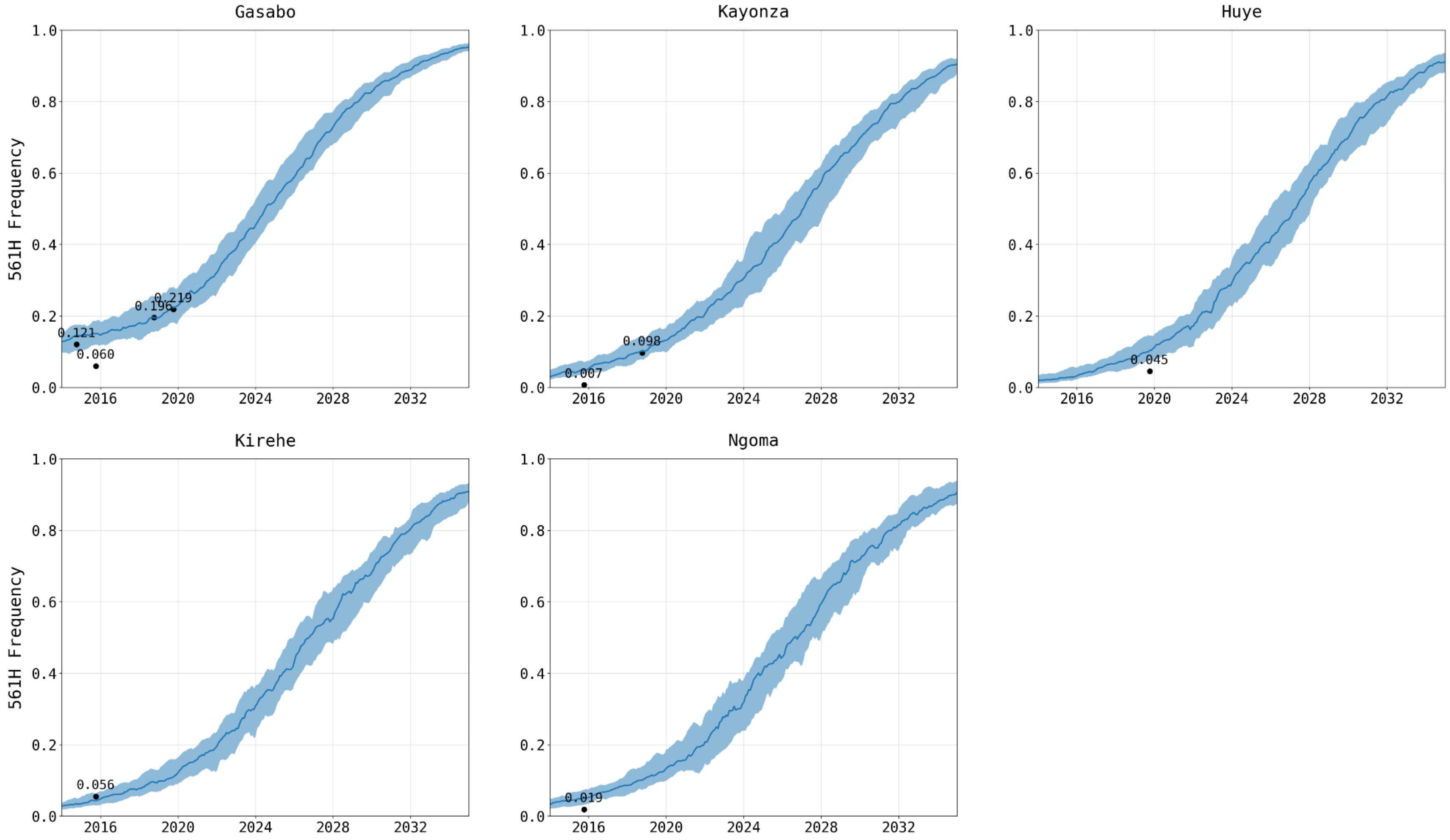

Figure S6. District level 561H frequency when the fitness penalty is increased to 25x the calibrated value and the introduction of 561H was through the one-time mutation of 50% of the infected individuals in Gasabo.

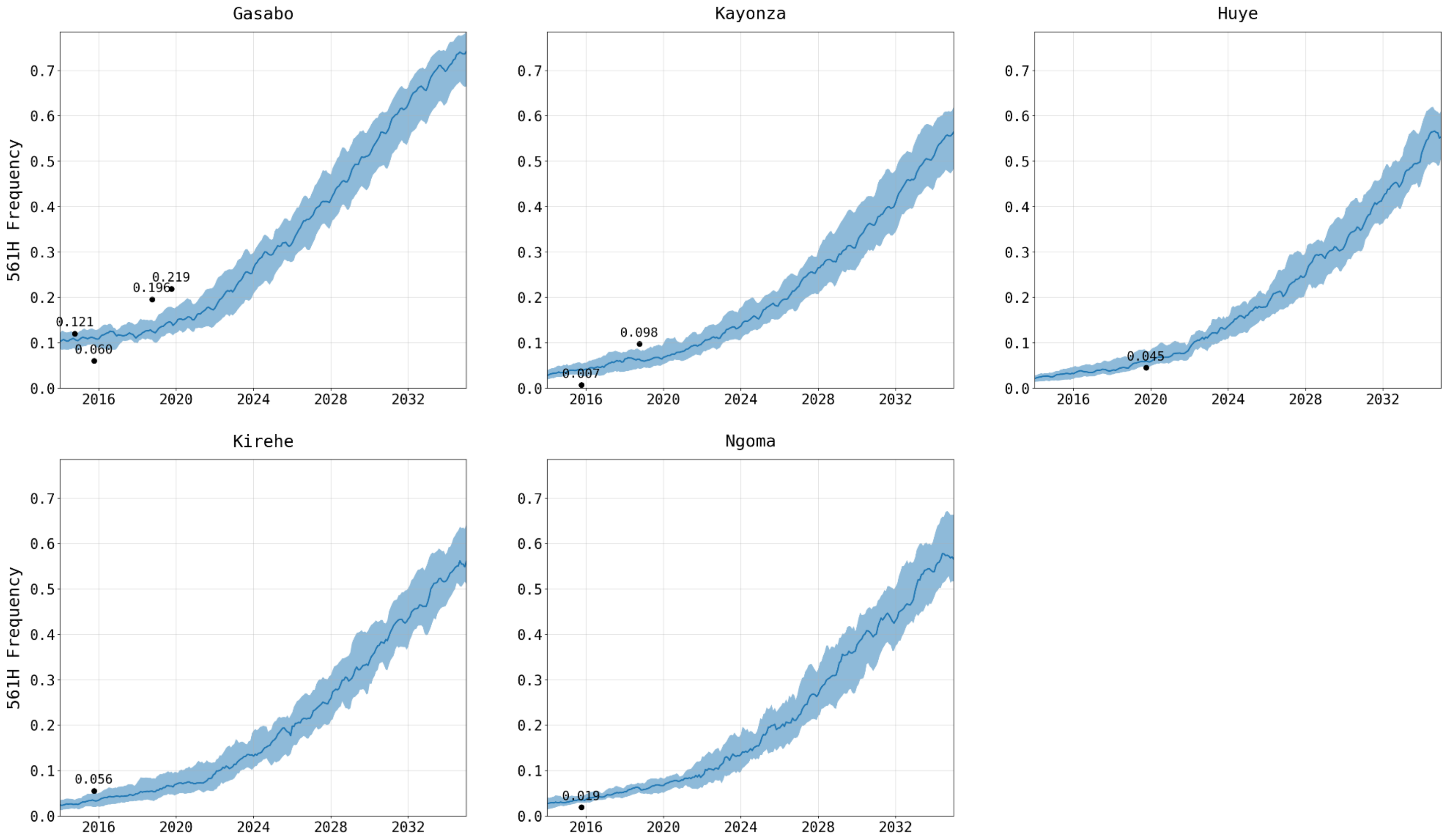

Figure S7. District level 561H frequency when the fitness penalty is increased to 50x the calibrated value and the introduction of 561H was through the one-time mutation of 75% of the infected individuals in Gasabo.

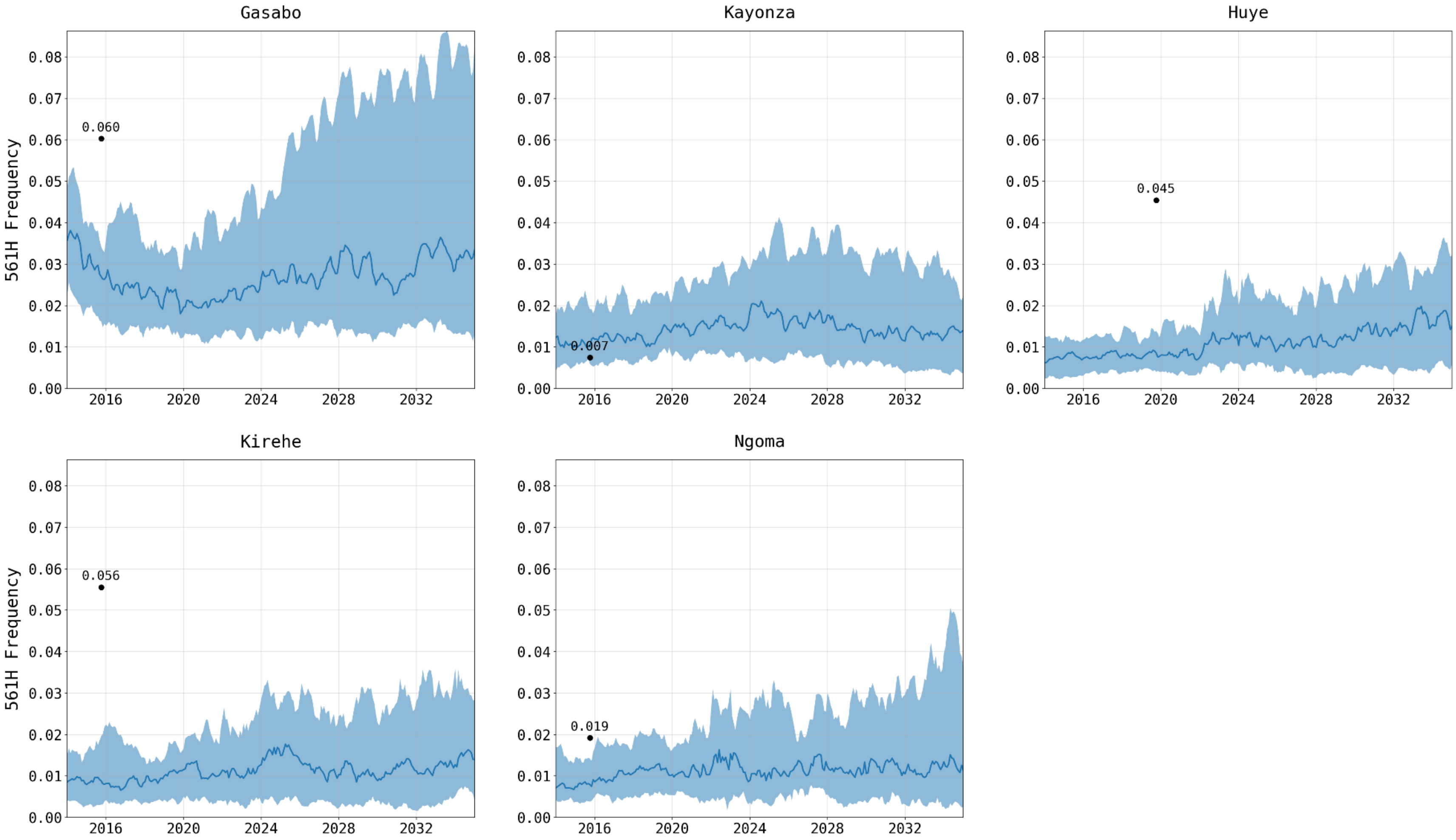

Supplement: Supplementary file 1 — Supplementary Tables 1–4 (concerning calibration parameters) and Figs. 1–7 (concerning model sensitivity analysis). [file 41591_2023_2551_MOESM1_ESM.pdf]
