# Supplementary material for: Modeling policy interventions for slowing the spread of artemisinin-resistant pfkelch R561H mutations in Rwanda
Source: Nat Med. 2023 Sep 21;29(11):2775–84. doi: 10.1038/s41591-023-02551-w (PMC10667088; doi:10.1038/s41591-023-02551-w)
Supplement: Supplementary file 2 — Reporting Summary [file 41591_2023_2551_MOESM2_ESM.pdf]

nature portfolio

Corresponding author(s): Robert Zupko, Maciej Boni

Last updated by author(s): Aug 7, 2023

Reporting Summary

Nature Portfolio wishes to improve the reproducibility of the work that we publish. This form provides structure for consistency and transparency in reporting. For further information on Nature Portfolio policies, see our Editorial Policies and the Editorial Policy Checklist.

Statistics

For all statistical analyses, confirm that the following items are present in the figure legend, table legend, main text, or Methods section.

n/a	Confirmed	
☐	☒	The exact sample size (*n*) for each experimental group/condition, given as a discrete number and unit of measurement
☒	☐	A statement on whether measurements were taken from distinct samples or whether the same sample was measured repeatedly
☐	☒	The statistical test(s) used AND whether they are one- or two-sided<br>*Only common tests should be described solely by name; describe more complex techniques in the Methods section.*
☒	☐	A description of all covariates tested
☒	☐	A description of any assumptions or corrections, such as tests of normality and adjustment for multiple comparisons
☐	☒	A full description of the statistical parameters including central tendency (e.g. means) or other basic estimates (e.g. regression coefficient) AND variation (e.g. standard deviation) or associated estimates of uncertainty (e.g. confidence intervals)
☐	☒	For null hypothesis testing, the test statistic (e.g. *F*, *t*, *r*) with confidence intervals, effect sizes, degrees of freedom and *P* value noted<br>*Give P values as exact values whenever suitable.*
☒	☐	For Bayesian analysis, information on the choice of priors and Markov chain Monte Carlo settings
☒	☐	For hierarchical and complex designs, identification of the appropriate level for tests and full reporting of outcomes
☒	☐	Estimates of effect sizes (e.g. Cohen's *d*, Pearson's *r*), indicating how they were calculated

Our web collection on statistics for biologists contains articles on many of the points above.

Software and code

Policy information about availability of computer code

Data collection	The simulation described (version 4.1.4) produced all data analyzed, which was aggregated in a Postgre (version 11.20) database, and downloaded into aggregated intermediate files using Python 3 (version 3.6.9). The intermediate files can be downloaded at https://github.com/bonilab/malariaibm-spatial-Rwanda-561H/raw/main/Data/rwa-intermediate.zip
Data analysis	The archived version the simulation code base (version 4.1.4), complied binaries for Linux, and scripts used for analysis and production of figures presented in this manuscript can be found on GitHub at https://github.com/bonilab/malariaibm-spatial-Rwanda-561H. ArcGIS Pro (version 3.1.2), MATLAB (R2021b, version 9.11.0.1809720 Update 1), and Python 3 (version 3.6.9) were used for data analysis and to prepare figures along with Adobe Illustrator 2023 (version 27.7) to combine and label figures.

For manuscripts utilizing custom algorithms or software that are central to the research but not yet described in published literature, software must be made available to editors and reviewers. We strongly encourage code deposition in a community repository (e.g. GitHub). See the Nature Portfolio guidelines for submitting code & software for further information.

Data

Policy information about availability of data

All manuscripts must include a data availability statement. This statement should provide the following information, where applicable:

- Accession codes, unique identifiers, or web links for publicly available datasets
- A description of any restrictions on data availability
- For clinical datasets or third party data, please ensure that the statement adheres to our policy

Intermediate data files produced by the simulation can be found on GitHub under https://github.com/bonilab/malariaibm-spatial-Rwanda-561H/raw/main/Data/rwa-intermediate.zip while the configuration files used for the study described in this manuscript can be found under https://github.com/bonilab/malariaibm-spatial-Rwanda-561H/tree/main/Studies. Global PfPR for children 2 to 10 years of age was used in calibrating the model, and the dataset was archived at https://github.com/bonilab/malariaibm-spatial-Rwanda-561H/tree/main/Data/GIS/MAP while the most recent data can be retrieved from https://data.malariaatlas.org/maps?layers=Malaria:202206_Global_Pf_Parasite_Rate. The spatial distribution of population is derived from the WorldPop 2015 spatial distribution of population in Rwanda, https://hub.worldpop.org/doi/10.5258/SOTON/WP00674

Human research participants

Policy information about studies involving human research participants and Sex and Gender in Research.

Reporting on sex and gender	Sex and gender are not included as part of the simulation and model calibration for treatment seeking and movement is based upon the aggregated population data that has been reported in the literature.
Population characteristics	N/A
Recruitment	N/A
Ethics oversight	N/A

Note that full information on the approval of the study protocol must also be provided in the manuscript.

Field-specific reporting

Please select the one below that is the best fit for your research. If you are not sure, read the appropriate sections before making your selection.

☐ Life sciences ☐ Behavioural & social sciences ☒ Ecological, evolutionary & environmental sciences

For a reference copy of the document with all sections, see nature.com/documents/nr-reporting-summary-flat.pdf

Ecological, evolutionary & environmental sciences study design

All studies must disclose on these points even when the disclosure is negative.

Study description	A mathematical modeling analysis -- using a previously calibrated, stochastic, individual-based model -- to evaluate 26 possible options for Rwanda's National Malaria Control Program to respond to the recent emergence and frequency increase of the artemisinin-resistant R561H allele in Plasmodium falciparum.
Research sample	No independent data were collected for this study. The study presents future projections of R561H evolution under 26 different treatment scenarios over the next 10 years.
Sampling strategy	N/A
Data collection	N/A
Timing and spatial scale	Simulation/project approaches start by matching Rwanda's (1) prevalance data, (2) incidence data, and (3) R561H frequency data from 2014 to 2022. This is done on a 5km-by-5km spatial scale in Rwanda.
Data exclusions	N/A
Reproducibility	All calibrations and simulation results are full reproducible, with source code and output files stored in a Github repository.
Randomization	N/A
Blinding	N/A

Did the study involve field work? ☐ Yes ☒ No

Reporting for specific materials, systems and methods

We require information from authors about some types of materials, experimental systems and methods used in many studies. Here, indicate whether each material, system or method listed is relevant to your study. If you are not sure if a list item applies to your research, read the appropriate section before selecting a response.

Materials & experimental systems

n/a	Involved in the study
☒	☐ Antibodies
☒	☐ Eukaryotic cell lines
☒	☐ Palaeontology and archaeology
☒	☐ Animals and other organisms
☒	☐ Clinical data
☒	☐ Dual use research of concern

Methods

n/a	Involved in the study
☒	☐ ChIP-seq
☒	☐ Flow cytometry
☒	☐ MRI-based neuroimaging
